# Diagnosis and biomarkers for ocular tuberculosis: From the present into the future

**DOI:** 10.7150/thno.81488

**Published:** 2023-04-01

**Authors:** Zhang Ludi, Ashita Ashish Sule, Ramar Perumal Samy, Ikhwanuliman Putera, Benjamin Schrijver, Paul Edward Hutchinson, Jayantha Gunaratne, Indu Verma, Amit Singhal, Rina La Distia Nora, P. Martin van Hagen, Willem A Dik, Vishali Gupta, Rupesh Agrawal

**Affiliations:** 1 Lee Kong Chian School of Medicine, Nanyang Technological University of Singapore, Singapore.; 2 Yong Loo Lin School of Medicine, National University of Singapore, Singapore, Singapore.; 3 National Healthcare Group Eye Institute, Tan Tock Seng Hospital, Singapore, Singapore.; 4 Department of Ophthalmology, Faculty of Medicine Universitas Indonesia - CiptoMangunkusmoKirana Eye Hospital, Jakarta, Indonesia.; 5 Laboratory Medical Immunology, Department of Immunology, ErasmusMC, UniversityMedical Centre, Rotterdam, the Netherlands.; 6 Department of Internal Medicine, Division of Clinical Immunology, Erasmus MC, University Medical Center, Rotterdam, The Netherlands.; 7 Department of Ophthalmology, Erasmus MC, University Medical Center, Rotterdam, The Netherlands.; 8 Immunology Programme, Life Sciences Institute, National University of Singapore, Singapore, Singapore.; 9 Institute of Molecular and Cell Biology (IMCB), Agency for Science, Technology and Research (A*STAR), Singapore, Singapore.; 10 Department of Biochemistry, Post Graduate Institute of Medical Education and Research, Chandigarh, India.; 11 A*SATR Infectious Diseases Labs (A*STAR ID Labs), Agency for Science, Technology and Research (A*STAR), Singapore, Singapore.; 12 Singapore Immunology Network (SIgN), Agency for Science, Technology and Research (A*STAR), Singapore, Singapore.; 13 University of Indonesia Hospital (RSUI), Depok, West Java, Indonesia.; 14 Advanced Eye Centre, Post-Graduate Institute of Medical Education and Research (PGIMER), Chandigarh, India.; 15 Duke NUS Medical School, Singapore, Singapore.; 16 Singapore Eye Research Institute, Singapore, Singapore.; 17 National Institute for Health Research Biomedical Research Centre, Moorfields Eye Hospital, London, UK.; 18 School of Pharmacy, Nantong University, Nantong, P. R. China.; 19 Department of Mechanical Engineering, University College London, London, United Kingdom.

**Keywords:** Ocular tuberculosis, molecular diagnostic techniques, biomarkers, multi-omic, precision medicine

## Abstract

Tuberculosis is an airborne disease caused by *Mycobacterium tuberculosis (Mtb)* and can manifest both pulmonary and extrapulmonary disease, including ocular tuberculosis (OTB). Accurate diagnosis and swift optimal treatment initiation for OTB is faced by many challenges combined with the lack of standardized treatment regimens this results in uncertain OTB outcomes. The purpose of this study is to summarize existing diagnostic approaches and recently discovered biomarkers that may contribute to establishing OTB diagnosis, choice of anti-tubercular therapy (ATT) regimen, and treatment monitoring. The keywords ocular tuberculosis, tuberculosis, *Mycobacterium*, biomarkers, molecular diagnosis, multi-omics, proteomics, genomics, transcriptomics, metabolomics, T-lymphocytes profiling were searched on PubMed and MEDLINE databases. Articles and books published with at least one of the keywords were included and screened for relevance. There was no time limit for study inclusion. More emphasis was placed on recent publications that contributed new information about the pathogenesis, diagnosis, or treatment of OTB. We excluded abstracts and articles that were not written in the English language. References cited within the identified articles were used to further supplement the search.

We found 10 studies evaluating the sensitivity and specificity of interferon-gamma release assay (IGRA), and 6 studies evaluating that of tuberculin skin test (TST) in OTB patients. IGRA (Sp = 71-100%, Se = 36-100%) achieves overall better sensitivity and specificity than TST (Sp = 51.1-85.7%; Se = 70.9-98.5%). For nuclear acid amplification tests (NAAT), we found 7 studies on uniplex polymerase chain reaction (PCR) with different *Mtb* targets, 7 studies on DNA-based multiplex PCR, 1 study on mRNA-based multiplex PCR, 4 studies on loop-mediated isothermal amplification (LAMP) assay with different *Mtb* targets, 3 studies on GeneXpert assay, 1 study on GeneXpert Ultra assay and 1 study for MTBDRplus assay for OTB. Specificity is overall improved but sensitivity is highly variable for NAATs (excluding uniplex PCR, Sp = 50-100%; Se = 10.5-98%) as compared to IGRA. We also found 3 transcriptomic studies, 6 proteomic studies, 2 studies on stimulation assays, 1 study on intraocular protein analysis and 1 study on T-lymphocyte profiling in OTB patients. All except 1 study evaluated novel, previously undiscovered biomarkers. Only 1 study has been externally validated by a large independent cohort.

Future theranostic marker discovery by a multi-omics approach is essential to deepen pathophysiological understanding of OTB. Combined these might result in swift, optimal and personalized treatment regimens to modulate the heterogeneous mechanisms of OTB. Eventually, these studies could improve the current cumbersome diagnosis and management of OTB.

## 1. Background of the clinical problems 1.1. Epidemiology of Tuberculosis and Ocular Tuberculosis (OTB)

Tuberculosis (TB), caused by *Mycobacterium tuberculosis* (*Mtb*), is a major public health problem, especially in developing countries. Currently the World Health Organization (WHO) estimates that one-quarter of the world's population is latently infected with *Mtb*, of these 10% will eventually progress to active tuberculosis 1-3. Furthermore, the WHO has estimated that 10 million people suffered from active TB in 2021, resulting in 1.6 million deaths 4. Despite the advancement of GeneXpert MTB/RIF, a nucleic acid assay for rapid TB detection that simultaneously tests for rifampicin sensitivity in pulmonary samples, extrapulmonary TB (EPTB) detection remains challenging due to pauci-bacillary specimens 5. In addition, suboptimal diagnostic performance of chest x-ray (sensitivity 87%, specificity 89%), sputum smear (sensitivity 32-94%, specificity 50-99%), culture (sensitivity 73-89%, specificity >99%), MTB/RIF (sensitivity 67-98%, specificity 98-99%) further complicates the diagnosis of pulmonary TB. Sputum smear, the classic method of detecting pulmonary TB, requires 5000 to 10,000 bacteria per ml for reliable detection. On top of mentioned assay limitation for the detection of TB only 5-10% of TB-infected individuals display clinical signs and symptoms associated with pulmonary TB 6. Currently available data on ocular tuberculosis (OTB) varies widely due to the lack of specific diagnostic criteria 7. Prevalence of ocular tuberculosis (OTB) on a global scale is estimated to account for 4.0% (95% CI 3.0-5.0%) of all uveitis cases and isreported to be one of the leading causes of infectious uveitis, ranging from 22.9-48.0% in Indonesiaand India 8-10. OTB can affect almost all parts of the eye and may cause sight-threatening complications, such as glaucoma, cataract, and cystoid macular edema in the absence of swift and appropriate treatment 11.

### 1.2. Clinical features and diagnostic challenges for OTB

OTB is an extrapulmonary manifestation of *Mtb* infection, which generally is transmitted through inhaled bioaerosols containing *Mtb* bacilli. Following inhalation, a primary infectious foci is formed in the lungs, dissemination can subsequently occur through the lymphatic and hematologic compartment resulting in EPTB, which can also include the eye's uveal tract (iris, ciliary body, and choroid) 12. It is hypothesized that *Mtb*-laden macrophages deposit in the first available capillary beds upon entering the eye, which most likely is the highly vascularized choroid, creating an oxygen rich environment analogous to the apex of the lung 13. These characteristics likely account for the fact that posterior uveitis is the most common presentation of OTB 14.

Apart from a secondary infection caused by haematogenous spread from a distant infectious site (i.e. lung), OTB can also occur as a primary infection in conjunctiva, cornea, sclera, adnexa, lids, and lacrimal apparatus 13. Due to the rarity of microbiological evidence of *Mtb* in ocular fluids two additional pathophysiological mechanisms of OTB have been suggested 15. The first one indicates that OTB could be a manifestation of a hypersensitivity response to *Mtb* antigens in the setting of local ocular infection 16. The second advocate that OTB is an autoimmune reaction to ocular antigens that results from antigenic mimicry between *Mtb* and ocular antigens (e.g. interphotoreceptor retinoid-binding protein-specific autoantigen), resulting in ocular inflammation even in the absence of mycobacterial products in the eye 17. How the various pathophysiological mechanism of OTB contribute to the disease and if typical clinical phenotypes can be attributed to a specific mechanism has not been clearly established yet. Despite *Mtb's* paucibacillary nature, a strong chronic and recurrent intraocular inflammatory response, often in need of adjunctive corticosteroid therapy, has been described. These observations heightened interest in immunopathogenic studies to identify novel biomarkers, such as proteins and metabolites, involved in disease activity and treatment efficacy 18.

OTB is considered as an imitator of various non-inflammatory and infectious or non-infectious inflammatory ocular pathologies, requiring a very high index of suspicion to be diagnosed 16, 19. Moreover, OTB can occur without clinically apparent pulmonary TB or other signs suggestive of active TB disease 20. Occult TB elsewhere in the body is generally restricted to paucibacillary and clinically silent sites such as the intrathoracic lymph nodes 18. Altogether, these difficulties contribute to diagnostic delays, which increase morbidity and vision loss.^19^

Recently, the current view of dichotomized “latent” and active “TB” has been challenged by the hypothesis that there are more than two different stages of TB: eliminated, incipient, subclinical form, and active TB 21. In ocular TB, many diagnostic confirmation relies on the positivity of immunoreactivity to TB antigens. A previous study found that even in histopathologically-proven ocular TB, active systemic TB may not be present in all cases 22.

As defined by WHO, a bacteriologically confirmed case of TB is whose biological specimen is positive by either smear microscopy, or culture or WHO-approved rapid diagnostic test. These tests fulfills the target product profile (TPP) criteria of goal sensitivity of ≥ 98% % in smear‐positive, culture‐positive pulmonary TB, and ≥ 68% in smear‐negative, culture‐positive adults 23. However, no optimal TPP diagnostic sensitivity for ocular fluid specimens have been recommended, although the revised recommended TPP for EPTB is 80% sensitivity for all forms of microbiologically-confirmed EPTB 24. Apart from this, even if a patient does not fulfil the criteria for bacteriological confirmed TB but has been diagnosed with active TB by a clinician, who then decided to initiate anti-tubercular therapy (ATT), is also considered as a clinically diagnosed case of TB. In this scenario the clinical diagnosis could be on the basis of Chest X-ray abnormalities or suggestive histology without laboratory confirmation 23.

On the other hand, global uveitis experts of the Collaborative Ocular Tuberculosis Study (COTS) group have agreed that there is currently no single gold standard diagnostic test for OTB. Clinical diagnosis of OTB is challenging due to highly variable clinical phenotypes, local prevalence of TB, immigration from a highly-endemic country and variable interferon-gamma release assay (IGRA), or tuberculin skin test (TST) or chest x-ray (CXR) findings 25. The key diagnostic criteria for OTB as defined by the Standardization of Uveitis Nomenclature (SUN) Working Group is a compatible uveitic syndrome, including: 1) anterior uveitis with iris nodules, 2) serpiginous-like tubercular choroiditis, 3) choroidal nodule (tuberculoma), 4) occlusive retinal vasculitis, and 5) in hosts with evidence of active systemic TB, multifocal choroiditis; including: 1) histologically- or microbiologically-confirmed infection, 2) positive IGRA test, or 3) positive TST 26. Putting all these together, the COTS group published an online, cost-effective, web-based clinical scoring system known as the COTS Calculator (https://www.oculartb.net/cots-calc) to guide the crucial decision of initiating ATT in clinically suspected OTB cases 27.

### 1.3. Corroborative tests for presumptive diagnosis of OTB

Confirmed OTB requires detection of *Mtb* from ocular samples. However, OTB is often paucibacillary and difficult to confirm by conventional tests such as nucleic acid amplification tests (NAAT), smear microscopy, or culture 18. Collection of ocular fluid (tears, aqueous humour, vitreous fluid) or retinal biopsy specimens to confirm the presence of *Mtb* in ocular tissue samples is not routinely performed due to the invasive nature of the procedures needed to acquire these ocular specimens which in addition form a potential risk for loss of visual acuity among patients with existing ocular inflammation 28. Therefore, clinical diagnosis of OTB is often presumptive, relying on indirect evidence of TB infection and exclusion of other possible uveitis causes. Corroborative tests like IGRA and TST and chest imaging, assessing the presence of TB suggestive lesions, are usually performed in the presence of a clinical OTB presentation and have a supportive role in the diagnostic workup 18.

Moreover, for most patients with TB-associated immune-induced retinal vasculopathy, actual detection of *Mtb* is uncommon because the yield of organisms from intraocular specimens is too low 19. The clinical presentation of tubercular retinal vasculitis can be variable, (1) as an exudative, segmental, hemorrhagic retinal vasculitis, usually associated with peri- or sub-vascular choroiditis, and vitritis, or (2) as a peripheral, minimally exudative, non-hemorrhagic, isolated retinal vasculitis with extensive (not segmental) swathes of pipestem-like sheathing with minimal or no vitritis. The latter is also referred to as “Eales disease” and is proposed to be more related to a hypersensitivity response to tubercular protein, hence proving the presence of *Mtb* may be difficult in this condition and the diagnosis is mainly based on immunoreactivity to TB antigen 14.

#### 1.3.1. Immunological skin and blood tests

TST also known Mantoux test is a century-old test to assess the presence of immunological memory against *Mtb* antigens (purified protein derivative (PPD)) during latent and active infection. It is low-cost and readily available in clinics worldwide. However, TST specificity is limited and may produce a false positive result in persons vaccinated with Bacillus Calmette-Guérin (BCG) or those infected with *non-tuberculous Mycobacterium (NTM)* 29. Of note, limitations of TST include false negative results in immunocompromised patients or populations with impaired cellular immunity, such as young children and the elderly 30. TST results can also be confounded by concurrent dermatological diseases such as psoriasis 18, 31. Moreover, significant variation in the administration and interpretation of TST affects the uniformity and objective reliability of the test. While the recommended cut-off diagnosis for latent TB infection (LTBI) is 10mm, this cut-off value is adjusted to more than 15mm in endemic countries to reduce the rate of false positives and over-treatment with anti-tubercular therapy 32. Overall, TST has a limited reported specificity of 51.1-85.7% and sensitivity of 70.9- 98.5% for diagnosing OTB (see Table 1).

IGRAs are full blood tests that asses IFN-γ production by T-lymphocytes in response to *Mtb* antigens, early secreted antigenic target 6 (ESAT-6) and culture filtrate protein 10 (CFP10). These antigens are encoded within the region of difference 1 (RD1) which is absent in BCG and most NTM. Hence, IGRA shows a better specificity for diagnosing OTB than TST (Table 1). The sensitivity of IGRA when compared to TST is more contentious with varying results in available reports. In five head-to-head comparisons of IGRA and TST in TB-endemic countries, IGRA sensitivity underperformed as compared to TST 33-36, while a non-comparative study by Ahn *et al*. reported a sensitivity of 100% 37. Amongst two head-to-head comparisons of IGRA and TST in non-endemic countries, only Llorenc *et al.* reported an improved IGRA sensitivity as compared to TST 38, 39. Overall, studies more often report a reduced IGRA sensitivity when compared to TST. In addition, the limited number of OTB studies, consistently reported underperforming IGRA sensitivities in TB-endemic countries. This is concordant with the WHO observation that IGRA sensitivity for TB is lower in TB-endemic countries 40. In practice, low-resources in most TB endemic regions, prevent the use of IGRA due to high cost, technical challenges and IGRA's inability to distinguish active from latent TB 33, 41. While current literature generally recommends IGRA as a routine screening tool for OTB, due to the low sensitivity of IGRA, negative or indeterminate IGRA results in patients with clinical characteristics strongly suggestive of OTB should be interpreted with caution as the presence of anti-IFN-γ autoantibodies might interfere with detectable IFN-γ levels 28. TST and IGRA tests demonstrate biomarker use in the form of indirectly and directly quantifying IFN-γ responses to aid OTB diagnosis.

#### 1.3.2. Radiographic tests

Other investigative tests include chest X-ray, fluorescein angiography, and ultrasonography. Chest X-ray is used to evaluate patients with suspected intraocular TB, since the lungs are most often the primary site of TB infection. Chest computed tomography and positron emission tomography scans are not routinely performed due to high costs, even though superior delineation between concomitant parenchymal, hilar, or pleural lesions in normal or inconclusive chest X-rays 44. However, chest radiologic evidence of post-inflammatory lesions such as parenchymal scarring or hilar lymphadenopathy is not specific to TB 45. Fluorescein angiography can assist in the evaluation of retinal vascular leakage and active choroidal lesions while ultrasonography can support differentiation between uveal tumors and tuberculomas 46.

**Figure 1:**
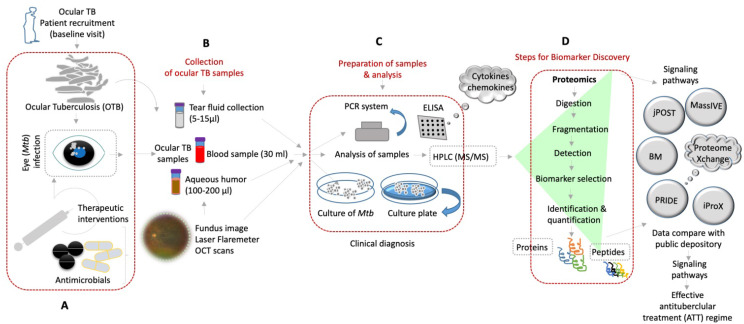
Shows the molecular diagnosis by various approaches of the ocular samples obtained from OTBpatients and identification of novel biomarkers for better treatment of ocular tuberculosis. (A-D) Diverse clinical approaches and diagnostic tools that are used to detect OTB from the patient's samples by using mass spectrometry based-proteomics/peptidomics and others for discovery of novel biomarkers (BM) of potential therapeutic targets and better treatment options of OTB.

As corroborative tests, IGRA/TST and chest radiography are insufficient to diagnose OTB. A previous study reported the presence of 1-2 *Mtb* bacilli at the level of the retinal pigment epithelium layer 22. This paucibacillary infection in RPE was sufficient to cause ocular inflammation, with some cases having a negative IGRA and TST underlining their ancillary nature 22. Novel molecular approaches in the diagnosis of OTB can be complementary to (rather than a substitute for) these existing and older tools 22, 47. Therefore, this review summarizes the published literature for existing confirmatory diagnostic tools in Section 2 and covers the technological development of molecular diagnostics using novel biomarkers for OTB in Section 3 (Figure 1).

### 1.4. Role of HIV co-infection on OTB diagnosis

An observational study conducted in a tertiary hospital in India reported elevated ocular morbidity (23.8%) in patients co-infected with TB and human immunodeficiency virus (HIV) as compared to those only infected with TB 48. Nevertheless, it is important to remember that studies on OTB diagnostic tests have largely been carried out in the HIV-uninfected populations, or the study designs do not distinguish patients with HIV from those without as the sample sizes are already small. Hence, most of the adjusted cut-offs in HIV patients come from studies observing HIV patients co-infected with other forms of EPTB, not restricted to OTB.

A significant proportion of HIV patients display CD4 counts <100 cells/μl. Therefore, a Mantoux test can underestimate the prevalence of TB when HIV patients have lower immune reactivity. The cut-off value for positive Mantoux therefore needs to be adjusted to 5mm for HIV patients unlike 10mm for immunocompetent patients 49. In a systemic review of 14 studies not limited to OTB patients, IGRA testing has a pooled sensitivity of 81% in the general population, but co-infection with HIV reduces sensitivity up to 63% 50. As HIV induces immunosuppression, extrapulmonary TB (EPTB) disease, mediastinal lymphadenopathy, and miliary tuberculosis become more common, whereas chest x-ray is less likely to show signs of upper lobe cavitation. PET/CT scans and unenhanced CT scans were found to both have similar sensitivity (33.3%) and specificity (100%) for EPTB in 29 OTB patients (5 were HIV-positive) 51. These findings clearly demonstrate that HIV infection affects interpretation of TB diagnostic tests. It would be prudent of ophthalmologists to check the HIV status of patients suspicious for OTB infection.

## 2. Existing diagnostic tools for OTB

### 2.1. Detecting *Mtb* using traditional bacteriologic tests

Traditional bacteriologic diagnosis used Ziehl-Neelsen or auramine-rhodamine staining's of ocular fluids or tissue sections to detect acid-fast bacilli (AFB) to diagnose OTB.^52^ Regarding pulmonary TB, acid-fast bacilli (AFB) smears in sputum samples display a sensitivity of 50% 53. Hence, the likelihood of detecting AFB in aqueous or vitreous fluid is even lower due to the paucibacillary nature of EPTB and intrinsic antimicrobial properties of conjunctiva and tears that further make the ocular surface paucibacterial with few culturable bacteria 19, 54, 55. Direct identification of *Mtb* by culture is considered the gold standard in diagnosis, however, only very few if any bacilli can be obtained from aqueous or vitreous humor and culture results may take 6-8 weeks, significantly delaying diagnosis and treatment initiation 52. Even though, costly semi-automated or fully-automated systems with liquid media can provide earlier results; diagnosis of OTB based on AFB smear and culture lacks accuracy and speed 53.

### 2.2. Significance of Molecular Signatures and Novel Biomarkers

**Figure 2:**
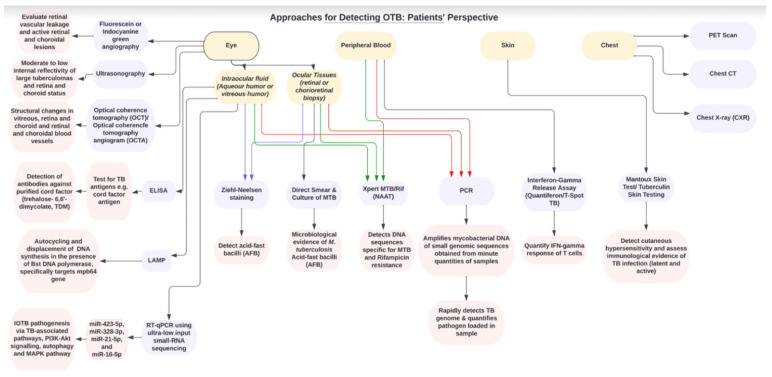
Flow chart showing the clinical approaches and diagnostic tools for detecting OTB from patient's perspective.

A biomarker is defined as a characteristic that is objectively measured and evaluated as an indicator of normal biological processes, pathogenic processes, or pharmacologic responses to a therapeutic intervention 56. Before diving deep into ongoing research on novel biomarkers, the following subsections aim to emphasize molecular and serological biomarker-based tools already used in clinical practice to directly identify *Mtb.* (Figure 2). These tests are not routinely performed as triage, unlike the corroborative tests described in the previous sections; however, they have been validated for clinical practice.

#### 2.2.1. Cellular and serological immuno-assays

It has been shown that antibodies against purified cord factor antigen, trehalose-6,6'-dimycolate (TDM), the most abundant cell wall component of *Mtb* bacilli, can be used for rapid serodiagnosis of pulmonary TB 57. A study by Sakai *et al* reported a sensitivity and specificity of 100% for the detection OTB by measuring anti-TDM IgG antibodies in serum samples of nine presumed OTB, three sarcoidosis and three Behcet's disease patients 58. However, validation studies with larger sample sizes are needed. A decline in anti-TDM antibody levels has been reported during pulmonary TB after ATT initiation, suggesting applicability to monitor treatment efficacy 59. Detection of anti-TDM antibodies suggests the presence of *Mtb* bacilli even in the absence of active systemic disease 58. However, as with IGRA and TST, detection of anti-TDM- antibodies does not differentiate between active, latent, or past TB infection (Figure 2).

#### 2.2.2. Detecting *Mtb* using nucleic acid amplification tests

Nucleic Acid Amplification Tests (NAAT) enables the amplification of pathogen-specific genomic DNA sequences from minute quantities of ocular fluid/tissue or blood samples. NAAT displays an improved sensitivity over traditional bacteriologic tests, smear microscopy and culture techniques described above (See Table 2). PCR, a form of NAAT, is increasingly commonly applied to rapid detect *Mtb*-specific DNA sequences such as IS6110 and MPB64 yielding an excellent specificity range of 80-100% (see table 2). However, *Mtb*-specific PCR performed on ocular fluid is relatively time-consuming and associated with a low to moderate sensitivity of 37.7-73.3%, resulting in false-negatives when either one of the IS6110 or MPB64 gene targets were used 60-64.

More advanced NAAT with improved diagnostic accuracy for detecting *Mtb* is also available. Multiplex PCR that is based on the amplification of three *Mtb* target genes (i.e. IS6110, MPB64, and rpoB or protein b) showed improved sensitivity (71.8-77.8%) compared to single-gene target PCR analysis due to the variable presence of IS6110 gene copy in North Indian populations, and false-negative detection of MPB64 gene in simultaneous *M. bovis* and *M. fortuitum* infection (see Table 2) 63-67. Another advantage of multiplex PCR is the possibility to test for multiple pathogens at once in very limited volumes of aqueous or vitreous fluid, which contributes to differential diagnoses.

Another type of NAAT, the loop-mediated isothermal amplification (LAMP) assay, targets specific *Mtb* genomic sequences to produce reliable results within 1 hour. The LAMP assay does not require expensive PCR instrumentation but rather uses *Bacilluss tearothermophilus* DNA polymerase under isothermal conditions (60-65ºC) in an ordinary laboratory water bath or heating block 64. This makes LAMP assay attractive to detect *Mtb,* especially in resource-limited settings. Based on DNA synthesis of the *Mtb* MPB64 gene, a 100% specificity and 67.1-85.7% sensitivity were reached in intraocular samples, while this was 100% and 64.3-70% respectively in the case of targeting the IS6110 gene 64, 68, 69. Sharma et al reported that IS1081, a multi-copy gene in the *Mtb* genome, has higher sensitivity (71.4%) than other single gene target LAMP (IS6110 and MPB64), while the multiplex LAMP consisting of all three gene targets yielded the highest sensitivity of 77.1% 64.

Although NAAT for *Mtb* detection in intraocular fluids displays a high specificity, the sensitivity is variable and limited. The latter may be explained by the following: (1) the limited (low) volume of ocular fluids and consequent minute quantity of genomic material obtained, (2) the non-uniform distribution of *Mtb* genomic material within specimens, (3) the presence of inhibitory factors within the specimens that interfere with NAAT efficacy, and (4) the lack of proper gold standard such that the diagnostic potential of NAAT is often evaluated against a presumptive OTB diagnosis 70. Data from the COTS-1 illustrated the clinical utility of PCR results. A positive PCR result may support the decision for ATT initiation to prevent the recurrence of OTB flare 47. Of note, a negative PCR result is insufficient to exclude OTB, and a decision to initiate ATT should be considered in conjunction with other corroborative test results and clinical presentation. In a recent meta-analysis, when the diagnostic value of the PCR result was calculated in relation to ATT treatment response in patients, the overall sensitivity and specificity were 88% (95% CI 83-92) and 71% (95% CI 60-80), respectively 71. This stresses the limited ability of the current PCR technique to diagnose ocular TB, even though it is still considered valuable in the diagnostic algorithm for OTB where it is available.

#### 2.2.3. Detecting drug resistance gene mutations using NAAT

Multidrug-resistant (MDR) OTB can be a reason for a worsening clinical presentation upon ATT initiation 72. Emerging molecular genetic tools are useful to rapidly detect drug resistance to ATT as opposed to drug sensitivity tests from bacterial cultures 73. Swift identification of drug resistance is critical to guide the proper choice of anti-tubercular treatment and to stop misuse of drugs that might perpetuate the evolution of additional drug resistance, which carries serious risks of MDR-TB transmission that is even more difficult to treat 74. While drug susceptibility testing using GeneXpert and Line Probe Assays (LPA), such as MTBDR plus, are well-documented in published literature for pulmonary TB, there is scanty evidence of ocular sample usage in LPAs.

The GeneXpert MTB/RIF assay is a WHO-endorsed, cartridge-based, automated assay based on real-time PCR or quantitative RT-PCR technology with a rapid turn-around time of 2-3 hours 63. It employs molecular beacons to detect *Mtb*-specific DNA and evaluate rpoB gene mutations responsible for rifampicin resistance. Even though, GeneXpert demonstrated a lower sensitivity of 10.5-17.2% in detecting *Mtb* from vitrectomy samples, it potentially provides rapid information on drug resistance in patients with OTB and is a recommended first-line screening test when available 63, 66, 67. In a large systematic review evaluating rifampicin resistance in adult sputum samples, GeneXpert pooled sensitivity for TB was 88% while in the HIV-positive sub-group, the sensitivity was only 80% 75. As this study did not evaluate ocular fluids in HIV patients with ocular inflammation, the sensitivity of GeneXpert in sputum samples of HIV patients seems to be much higher than the sensitivity in vitreous samples of OTB patients with unknown HIV status (see Table 2).

MTBDR plus assay performed on vitreous fluid samples could detect rifampicin resistance based on mutations in the rpoB gene and isoniazid resistance based on mutations in the katG and inhA genes, with a reported sensitivity of 34.5% and specificity of 100% 63. Probes detecting wild-type and mutant variations of these genes are added as part of the quantitative RT-PCR technique, which has a turnaround time of 24 hours, thus making the MTBDR plus assay an effective alternative to gene sequencing for the proper diagnosis of MDR-TB especially in isolated-rifampicin resistance regions.

With the rapidly expanding field of systems biology, where computational and mathematical analysis of large genomic and/or proteomic datasets are utilized to model complex biological systems, new mechanistic insights into the pathogenesis of TB as well as the discovery of novel biomarkers have been recently obtained. These discoveries can help to optimize diagnosis and development of personalized TB treatment tailored to specific host and/or pathogen factors 76. Next-generation sequencing (NGS) technologies that generate comprehensive TB drug-resistance profiles will likely be the first-line diagnostic tool to guide early initiation of ATT 77. In coming years, genomic technologies may detect particular mutations in the mycobacterial genome linked to resistance or unresponsiveness to ATT resulting in disease relapse or treatment failure 78

## 3. Novel biomarkers for diagnosis, treatment effectiveness, and prognostication of OTB

**Figure 3:**
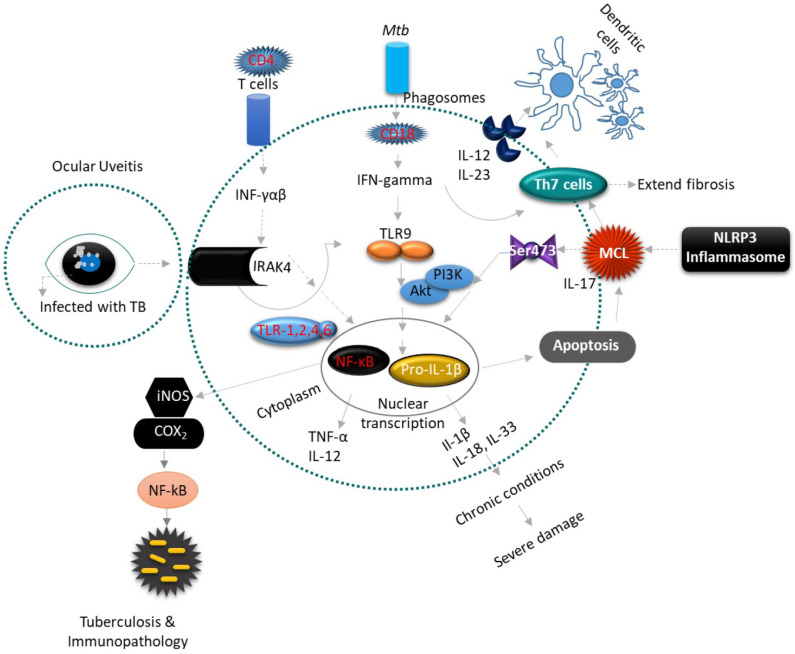
Uveitis is a form of eye inflammation that affects the middle layer of tissue in the eye wall (uvea). It presents with eye redness, pain, blurred vision and deterioration of visual acuity occurs quickly. (A-B) Inflammatory cytokine Interleukin 1 (IL-1) is an essential mediator of innate immunity and promotes inflammatory tissue damage in ocular uveitis (OU). The inflammatory constituents include Th1 and Th17 lymphocytes, which produce pro-inflammatory cytokines such as IL-1β, IL-2, IL-17, IL-18, IL-23, iNOS, COX2, TNF-α and INF-gamma that recruit leukocytes from circulation to result in tissue damage of the eye. The IFN-gamma-driven antimicrobial properties of phagocytes are augmented by IL-18 and IL-1β, and inflammatory cytokines processed by caspase-1, which are recruited to the inflammasomes. Inflammasomes are multimeric protein complexes that serve as a platform for caspase-1, the enzyme responsible for proteolytic cleavage of IL-1β and IL-18 precursors. However, this inflammasome activation triggers the multifaceted action of pro-inflammatory cytokines, a prerequisite for developing an effective inflammatory response against *Mtb.* The NLRP3 and AIM2 inflammasomes play an important role in innate immunity against *Mtb.*

As described above none of the available tests, especially in light of OTB, displays optimal diagnostic accuracy, therefore, novel biomarkers are still needed to optimize diagnosis and predict or monitor treatment response and outcome. The following section will discuss the role of novel discovered biomarkers in the pathogenesis, disease activity, and modulation of OTB. In addition, molecular mechanisms within the ocular immune system in response to OTB will be discussed (Figure 3). These novel biomarkers have not yet found their way into a clinical application, as they need to be validated to show accuracy in distinguishing individuals with OTB from patients with recurrent ocular inflammation. Point-of-care (POC) diagnostic tests to incorporate novel biomarkers in the real-life settings are also still being developed. Nonetheless, novel biomarkers discovery might identify new therapeutic ocular targets that contribute to improved diagnosis and personalized OTB treatment regimens, thus avoiding the use of unnecessary drugs and reducing systemic side effects 85. In this section, the advantages and disadvantages of each omic approach has been analyzed (see Table 3), and a further breakdown of the insights and limitations of each OTB novel biomarker study has been presented (see Supplementary Table 1).

### 3.1. Mycobacterial genomics: Future improvements

In a previous section on NAAT, different existing techniques for *Mtb* detection and drug-resistance screening have been discussed. Validation of resistance prediction is a crucial step to bring NAAT into clinical practice for pulmonary TB, as is the case for OTB in certain endemic or resource-sufficient regions 86, 87. However, even in the treatment of pulmonary TB, routine and full-scale implementation of NAAT-based drug-resistance profiling into clinical and public health practices is hampered by low bacterial loads and possible contaminations in sputum specimens 88, 89. Additional limitations include the lack of standardized genetic markers indicative of drug resistance that warranting induction of a personalized ATT and high laboratory costs associated with NAAT implementation 90. In this section, we discuss the possible solutions to overcome these limitations with the aim of NAAT *Mtb* genome analysis to assess treatment response, risk of relapse and/or treatment failure of OTB.

Targeted sequencing of *Mtb* genes associated with drug-resistance, as opposed to whole genome sequencing might improve diagnostic limitations due to limited recovery of *Mtb* DNA from obtained clinical samples. This is especially relevant in the case of paucibacillary infection usually observed in OTB 91. In recent years, the abundant cleavage activities of activated viz. Cas (CRISPR-associated proteins) has been harnessed for *in vitro* diagnosis of viral pathogens (e.g. zika, dengue) 92. As part of the adaptive immune systems of bacteria and archaea, CRISPR-associated proteins can detect target DNA molecules at concentrations as low as 5aM^10^which makes them excellent diagnostic tools for detection of (paucibacillary) infections 93-97. A rapid CRISPR-based assay for *Mtb* detection from various clinical samples was developed and its diagnostic performance was compared to *Mtb* cultures and GeneXpert MTB/RIF assay 98. The study found that CRISPR-MTB had a higher sensitivity of 79% as compared to 33% and 66% of culture and GeneXpert, respectively 98. CRISPR-MTB allowed for near single-copy sensitivity without compromising specificity (98%). The test requires less sample input and a shorter turnaround time for TB diagnosis and drug resistance compared to current clinical used test 98. The applicability of CRISPR-MTB technology on ocular fluid samples for OTB remains undetermined, warranting clinical evaluation.

However, partial characterized *Mtb* mutations still require culture-based drug-susceptibility phenotyping to determine the minimum inhibitory concentrations (MIC) of ATT drugs needed for optimal personalized therapy. Hence, there is increasing interest in “big data” analysis of genotypic and phenotypic datasets to improve the accuracy of resistance predictions in MDR TB 76. Machine learning algorithms and genome-wide association studies are often employed to determine the phenotypic impact of nonstandard mutations to new and repurposed second-line drugs such as bedaquiline, clofazimine or linezolid to which resistance is not yet widespread 99, 100. These developments can guide future direction of personalized ATT regimens for OTB patients who may also contract these increasingly common and problematic MDR *Mtb* strains.

### 3.2. Human and mycobacterial transcriptomics

Among the host-based biomarkers, microRNAs (miRNAs) have emerged as an important candidate to diagnose infectious diseases, including TB 101, 102. Chadalawada *et al*. showed that four miRNAs, miR-423-5p, miR-328-3p, miR-21-5p, and miR-16-5p, were significantly dysregulated in aqueous humor of OTB patients 103. These four miRNAs could contribute to the pathogenesis of OTB via tuberculosis-associated pathways like phosphatidylinositol 3-kinase protein kinase B (PI3K-Akt) signaling, autophagy, and the mitogen-activated protein kinase (MAPK) pathway 103.

Human and *Mtb* transcriptomic insights obtained from pulmonary TB studies, might be of value in the context of OTB after validation. Firstly, changes in *Mtb's* transcriptome in response to 75 different anti-TB agents was evaluated by RNA sequencing and related to the mechanism of action of each agent 104, 105. Secondly, dual RNA sequencing allows simultaneous and unbiased profiling of human and *Mtb* transcription by capturing the transcriptome in its entirety, hence allowing a deeper understanding of the molecular host-pathogen interaction during *Mtb* infection 106, 107. Thirdly, highly sensitive (10 CFU/mL^-1^) detection of 16S ribosomal RNA (rRNA) might be applicable to monitor treatment response, even after weeks of ATT treatment 108. Since 16S rRNA is more stable and abundant than mRNA, 16S rRNA might be a superior biomarker to detect the presence and quantity of *Mtb* bacilli in ocular fluid specimens 109. Lastly, there is an increasing number of combination host RNA gene signatures from whole blood published in the literature for pulmonary TB 110-114. None of these transcriptomic signatures have been implemented into routine clinical practice so far 115.

Importantly, many of the pulmonary TB biomarker genes have been shown to relate to interferon (IFN) signaling 116, 117. Type I IFN-signaling has been shown to be involved in the pathogenesis of TB based on the observation of neutrophil-driven IFN-inducible gene profiling 118. Type 1 IFN contributes to the death of *Mtb*-infected macrophages, which might potentiate subsequent *Mtb* spread to the other cells 119. In depth understanding of IFN signatures might provide additional options to distinguish between active and latent TB. Profiling of IFN-inducible genes has been done in the context of uveitis of unknown cause with a positive QFT-Gold In-Tube test. La Distia Nora *et al.* proposed a whole blood IFN signature based on 10 interferon-stimulated genes as an applicable tool to stratify QFT-positive patients with uveitis of unknown cause into groups of high and low risk of having active TB-associated uveitis 120. Schrijver *et al.* further evaluated that the peripheral blood type 1 IFN gene signature score displayed an inverse correlation with serum complement component C1q. Combined measurement resulted in improved identification of ocular TB from uveitis with coincidental QFT positivity yet without other signs of active TB infection especially in high TB-endemic regions 121. In addition to multiple studies showing an important role of IFN signaling in the pathogenesis of pulmonary TB 122, 123 an *in vitro* study also displayed strong IFN signaling in RPE cells after *Mtb* infection 124.

Overall, miRNA targets, rRNA targets and Type 1 IFN gene signatures are emerging as potential biomarkers involved in OTB pathogenesis pathways to confirm the presence and activity of *Mtb* infection in the eye. However, these markers remain in the nascent trial phase and have yet been externally validated with a large independent cohort.

### 3.3. Proteomics: Human inflammatory cytokines and chemokines

Proteins, peptides, and protein post-translational modifications are robust biomarkers for early disease detection, disease classification, patient stratification, diagnosis, prognosis, and even monitoring disease activity and treatment efficacy 125. Advanced mass spectrometry (MS)-based clinical proteomics has emerged as a powerful and influential technological platform for the confident identification of such markers in complex biological samples 125. Proteomic analysis presents the opportunity to identify novel theranostic biomarkers with the potential for targeted therapy in the ocular field 126.

Vitreous proteomics is a rapidly emerging and promising field aimed at advancing the diagnostics and therapeutics of various debilitating ocular diseases 127-130. For example, a recent study showed discovery of novel therapeutics targets for diabetic retinopathy through systematic analysis of vitreous proteomics 131. Another study based on vitreous proteomics reported that elevated levels of extracellular carbonic anhydrase-I (CA-I) in vitreous from individuals with diabetic retinopathy that led to develop plasma kallikrein (PKal) inhibitors as a potential treatment for DME 132, 133. Proteomics analysis on various intraocular structures other than vitreous humour, such as aqueous humour,tears, cornea, lens, ciliary body, retina and retinal pigment epithelium can also be utilized to gain valuable insights into complex molecular signaling pathways of OTB. In addition, a combination of several biomarkers, a so-called disease-defining biosignature, can render a more accurate diagnosis of OTB. The following subsections compile the published literature wherein proteomics was performed on aqueous and vitreous humour and serum. We also explore future possibilities of tear proteomics in OTB.

#### 3.3.1. Ocular fluid proteomics for OTB

Several studies reported protein analysis from aqueous and vitreous humor in OTB patients. Ang *et al.* performed an aqueous humour cytokine and chemokine analysis in tubercular anterior uveitis patients and reported a significant increase in inflammatory cytokines such as IL-6 and CXCL8/IL-8 and Th1 associated chemokines CXCL9, and CXCL10 134, which is more consistent with an autoimmune-related ocular inflammation triggered by *Mtb* rather than an active ocular tuberculous infection 134. De Simone *et al*. recently studied the cytokine profile of the aqueous humour and discovered a potential biosignature that can guide more accurate diagnosis and treatment of difficult overlapping cases such as ocular sarcoidosis (OS) and OTB which share remarkably similar epidemiology, pathogenesis, and ocular presentations 135-137. Thirty-two patients, 15 with OS, five with QFT-positive OS, and 12 with presumed OTB, had blood and aqueous humour samples collected pre-treatment for the analysis of selected cytokines 135. Results confirmed that CXCL8, CXCL10, and IL-6 levels were higher in aqueous humour samples than in peripheral blood of all three patient groups, suggesting an intraocular source of cytokine/chemokine production which could serve as a localized therapeutic target for both OS and OTB. Aqueous humour CXCL8, CXCL10, and CXCL13 levels were significantly higher in definite OS than in presumptive OTB. However, there were no statistically significant differences in terms of cytokine levels among QFT-positive OS as well as presumptive OTB sample groups. These two sample groups showed similar aqueous humour levels of CXCL9, CXCL10, IFN-γ, IL-2, and IL-15. On the other hand, definitive OS samples showed prevalent aqueous humour expression of CCL20/ MIP-3α, CXCL13, and IL-10. These observed differences in aqueous humour and serum chemokine profiles could contribute to differentiate patients with OS from patients with OTB or concurrent OS and OTB as these display overlapping clinical phenotypes of granulomatous uveitis 135. These findings are in line with a previous study that reported elevated CXCL8/IL-8 and CXCL10 levels in aqueous humour samples of presumed OTB patients 138.

These recent findings highlighted the use of human vitreous proteomic analysis to differentiate many forms of intraocular inflammation, including OTB. Several differentially-expressed proteins could help differentiate granulomatous uveitis from other entities, along with the usefulness of vitreous humour CCL17 and CXCL13 to distinguish OTB from sarcoidosis 139. A recent study showed significantly reduced vitreous humour levels of CXCL13 in OTB as compared to OS and QuantiFERON-TB Gold-positive OS 135. Future extensive analysis of the vitreous phospho-proteome may represent another level of protein analysis worth exploring to gain additional pathophysiological insight into OTB and reveal potential novel biomarkers 140.

Bansal *et al.* analyzed disease-specific protein biosignatures in vitreous samples of 13 OTB patients. They found that OTB patients showed 11 upregulated proteins and 21 downregulated proteins compared to the combined non-TB uveitis and non-uveitis groups 141. The upregulated proteins included insulin-like growth factor 2 messenger RNA binding protein 3 (IGF2BP3), Complement component C8 beta chain (C8B), and other proteins involved in complement activation and the coagulation cascade. The downregulated proteins included Glucose-6-phosphate isomerases (GPI) and other proteins involved in carbohydrate-metabolism, gluconeogenesis, and glycolysis. A sub-group analysis showed that a signature consisting of 21 upregulated proteins (related to apoptosis and KRAS signaling), along with 37 downregulated DEPs (related to mTORC1 signaling, gluconeogenesis, and glycolysis) differentiated OTB from non-OTB 141. Future in depth validation and interpretation of the identified DEPs and associated pathways are needed to confirm the signatures robustness and accuracy and evaluate the potential implementation as a POC test 141.

#### 3.3.2. Serum proteomics for OTB

To our knowledge, there is only one published study on serum proteomics in OTB. Van de Colff *et al.* conducted a small pilot study comparing the levels of 29 known potential candidate biomarkers for pulmonary TB in serum and urine samples 142. Most biomarker concentrations were significantly higher in serum than in urine, hence these 2 biofluids cannot be used interchangeably when studying biomarker profiles in future 142. Although the study found that 2 biomarkers (IL-1RA and IL-2) showed higher concentrations in urine than serum and that three biomarkers (sIL-2Ra, sTNFRI and IFNγ) showed no difference in concentration between urine and serum, this study is not conclusive for candidate biomarkers specific for OTB as there is no negative control group 142.

Three studies described the potential of serum proteomics analysis in pulmonary TB. Mateos *et al.* showed that C-reactive protein (CRP), haptoglobin (HPT) alpha-1-acid glycoprotein 1 (A1AGP1), complement component C9 (C9), neutrophil defensin 1 (DEF1), and serum amyloid P component (SAA2-4) were elevated where apolipoprotein A (APOA1 and 2), serotransferrin (TRFE) and plasma kallikrein (KLK1B) were significantly decreased in active TB patients as compared to LTBI and healthy controls. Each of the markers: CRP, A1AGP1, KLKB1, TRFE, or APOA1 had an area under the curve value >70% 143. Another study by Peng *et al.* determined the presence of serum antibodies against 100 different *Mtb* antigens. Antibody levels against 15 *Mtb* antigens were significantly elevated and could distinguish between active and LTBI: MT1560.1-IgM, Rv0049-IgM, Rv0270-IgM, Rv0350-IgG, Rv0350-IgM, Rv0494-IgM, Rv1597-IgM, Rv1860-IgG, Rv1876-IgM, Rv2031c-IgG, Rv2352c-IgM, Rv2450c-IgM, Rv2511-IgG, Rv2688c-IgM, and Rv3480c-IgM. Combining the 15 markers to differentiate active TB from LTBI resulted in a good sensitivity of 85.4% and specificity of 90.3% 144. Future studies on OTB patients are needed to reveal whether serological proteomes could identify OTB biomarkers and provide pathophysiological insights. Garay-Baquero *et al.* reports a comprehensive TB plasma proteome by profiling 5022 proteins with diverse biochemical and molecular properties 145. Novel candidate biomarkers (CFHR5, ILF2) were verified in two independent cohorts, leading to the development of a 5-protein biosignature (CFHR5, LRG1, CRP, LBP, and SAA1) capable of discriminating TB from other respiratory diseases (AUC = 0.81) 145.

#### 3.3.3. Possibility of tear proteomics for OTB

Tears are attractive biofluids to study uveitis with two major advantages: tears are localized to the source organ, and tears are easily obtainable due to the non-invasive nature of sample collection. Hence, tear-proteomic profiling provides an attractive and non-invasive opportunity to discover novel biomarkers associated with pathologies of the ocular surface (e.g. dry eye diseases), posterior eye (e.g. diabetic retinopathy, age-related macular degeneration) and systemic diseases (e.g. Alzheimer's dementia, Parkinson's disease, multiple sclerosis, breast cancer) 126, 146. Tear proteome profiling in keratoconus patients identified 58 proteins related to process of cell death, oxidative damage and inflammation specific to tears of patients with keratoconus; 41 proteins involved in iron pathways was found to be underexpressed in keratoconus patients and identified in control samples only 147. Besides improving the knowledge of the disease's pathophysiology, Goni *et al.* also analysed the tear proteome profile before and after treatments and identified biomarkers, mainly A-kinase anchor protein 13 (AKAP-13), altered by surgical treatment 148. O'Leary *et al.* discovered a panel of 13 discriminant tear protein markers to distinguish mild ocular graft versus host disease to moderate-to-severe disease 149. Also, the use of novel targeted proteomic approaches are of interest to unravel disease-specific tear proteomes, such as in non-infectious uveitis (our data, unpublished) 150. Although extensive tear-proteome profiling data in OTB is still not provided in the current literature, we believe that such studies are warranted as these would contribute to improved diagnosis in material that can be non-invasively obtained, thereby contributing to patient well-being 126.

#### 3.3.4. Future of proteomics for OTB

Plasma and sputum proteomics allowed differentiation between active and latent pulmonary TB 151, 152. Protein signatures, including those in stimulated whole blood cultures and sputum samples, could also help distinguish MDR from drug-susceptible pulmonary TB 153-155. Increased levels of serum matrix metalloproteinases were also shown to be associated with disease severity 156. In addition, distinct proteomic signatures found in bronchoalveolar lavage fluid can also stratify treatment response by distinguishing clinically cured pulmonary TB patients from patients with active pulmonary TB 157. These obtained results in the light of pulmonary TB pave the way for future directions of proteomic studies investigating aqueous-and vitreous humour and tears from OTB.

### 3.4. Possibility of metabolomics for OTB

Only a few studies explored the potential of metabolites in blood, urine, or cerebrospinal fluid as biomarkers for pulmonary TB progression and treatment response 158-161. The level of seryl-leucine core 1 O-glycosylated peptide (SLC1G) in urine was significantly higher in TB patients than household contacts, and decreased in ATT responders 161. Hence, SLC1G could be a potential POC biomarker to monitor treatment response which needs further validation. Another study associated low tryptophan cerebrospinal fluid levels with improved survival outcome in TB meningitis 160.

Due to the blood-aqueous and blood-retinal barriers, it is assumed that eye tissue has its own distinct metabolic regulation 162. Hence, the metabolome of aqueous and vitreous humour in OTB may represent the local metabolic environment and may vary greatly from metabolomes of other bodily fluids present in systemic TB. Metabolomics has been used to analyze healthy ocular fluids, study tissue metabolism, and assess the effect of radiation on the eye 162, 163. Metabolomics from various sources, including aqueous humor, tears, and retina, have also been studied in other ophthalmic diseases such as keratoconus, glaucoma, and diabetic retinopathy using nuclear magnetic resonance (NMR) spectrometry and liquid and gas chromatography MS techniques 164-166. Metabolome studies in intraocular fluids of patients with OTB will be an exciting next step to understand *Mtb* pathogenesis and identify novel OTB-associated biomarkers.

### 3.5. Stimulation assay

Makhoba *et al.* recently assessed the diagnostic potential of previously identified biomarkers in the context of OTB for the first time by measuring 47 host biomarkers in a blood sample of 92 patients after stimulation with *Mtb* antigens (i.e. ESAT6, CFP10, and TB7.7) which are used in the QuantiFERON (QFT) In Tube test 167. Fourteen of these host derived biomarkers showed a significant difference between patients with probable and possible OTB versus those patients with other ocular diseases. The most promising individual biomarkers for diagnosing OTB and excluding other diseases are CCL4/MIP-1β, CCL8/MCP2, IFN-γ, IL-2, IL-22, and CD258 (LIGHT) which were shown to have sensitivities of less than 50% and specificity approaching 98%. The most accurate four-analyte biosignature included CD40L, IFN-γ, IL-33, and serum amyloid P (SAP), which combined had a sensitivity of 56.3% and specificity of 90%. Biomarker signatures can be further optimized to increase the sensitivity and specificity and ensure identification of patients misclassified by an individual marker 167. We noticed that this study was performed in South Africa, a TB-endemic country where a lot of patients may be latently infected with *Mtb* and thus will harbor T-lymphocytes that recognize *Mtb* antigens. Hence, cells from these latent-infected subjects would secrete host biomarkers after QFT-antigen stimulation even in the absence of active ocular or systemic infection 167.

A study by Alam *et al*. demonstrated evaluated intracellular cytokine responses by CD4^+^ T-lymphocytes isolated from vitreous, when stimulated with ESAT-6 and IRBP (interphotoreceptor retinoid-binding protein) peptides. They used the recently published SUN classification criteria for OTB, which divided patients into three groups: OTB, undifferentiated with IGRA/TST-positive (unknown), and controls. An increased percentage of IL-17 positive vitreous infiltrating CD4^+^ T cells was detected in the OTB group upon stimulation with ESAT-6 while double positive (i.e. IFNγ^+^IL-17^+^, TNFα^+^IL-17^+^, and TNFα^+^IFNγ^+^) vitreous infiltrating T cells were significantly more abundant in the unknown group. However, in all groups, TNF-α, IL-17A, and IFN-γ production upon stimulation with IRBP peptides were comparable 168. This suggested the applicability of utilizing vitreous T cell responses as a good biomarker for confirming OTB, but could be challenging in a routine clinical lab.

### 3.6. Serum and intraocular protein analysis

Recently, we described serum CCL17 as a biomarker with the potential to support the stratification of OTB from OS and QFT-negative uveitis. Consistent findings were also reported from TB and sarcoidosis patients in general and thus warrant further exploration to prove clinical applicability 169. Some reports suggest combining serum measurement of IFN-α, IL-10, and IL-2 with other cytokines like TNF-α, thereby improving the discriminating potential between active and LTBI 170, 171. TNF-α is also possibly a key marker in the reactivation of OTB when immunosuppressive anti-TNF-α agents are used for other confounding or concurrent ophthalmic inflammatory conditions 172. The phenomenon of paradoxical worsening in both OTB and pulmonary TB presents as a continued progression of TB lesions despite ATT initiation with unclear pathophysiology. A plausible explanation for this phenomenon includes the release of TNF-α and IL-1 by activated macrophages and monocytes exacerbating inflammation, which indicates increased immunosuppression with steroids and continuation of ATT 173. Another study tried to measure serum type-1 IFN levels but did not identify a difference in IFNα or IFNβ levels comparing LTBI to active TB, suggesting a discordance between type-1 IFN-induced RNA expression in peripheral blood leukocytes and circulation IFN levels in TB 174.

Several studies explored the potential of vascular endothelial growth factor (VEGF) and fibroblast growth factor (FGF) measurements from intraocular fluid samples. VEGF is crucial in angiogenesis and lymphangiogenesis as it is induced in response to tissue inflammation and hypoxia 175. Thayil *et al.* showed in guinea pig ocular models that hypoxia within TB granulomas was directly related to the extent of inflammation and associated with vascular occlusion resulting in reduced lesional oxygen tension 176. This drives excessive VEGF production within the inflamed granulomatous ocular tissues. Singh *et al.* demonstrated raised VEGF and decreased FGF vitreous humour levels in samples obtained from TB uveitis patients, and in supernatant of human RPE cell cultures infected by *Mtb* 177. Clinically, intra-vitreal anti-VEGF therapy was found beneficial in TB granuloma treatment in some case reports. This treatment response was associated with a reduction in granuloma vascularity, causing caseation and tissue necrosis of the granulomatous lesions 178, 179. *Mtb* induced suppression of the FGF pathway may represent a host immune evasive mechanism and enable *Mtb* proliferation 180. In this regard the role of FGF in the pathogenesis of *Mtb-*induced ocular inflammation and its application as a biomarker or therapeutic target in OTB remains to be explored.

### 3.7. T-lymphocyte profiling

TB-related immunity is primarily cell-mediated, involving IFN-γ production by CD4^+^ and CD8^+^ T-lymphocytes resulting in macrophage activation and improved intracellular *Mtb* killing 49. The role of humoral immunity in TB pathogenesis remains obscure. In pulmonary TB, T-lymphocyte-related biomarkers have been identified to distinguish active from LTBI 181. At present, increased membrane expression of CD38 and HLA-DR 182 by *Mtb-*specific CD4^+^ T-lymphocytes (HLA-DR^+^IFNγ^+^, CD38^+^IFNγ^+^) 183, or other CD4^+^ T-lymphocytes such as TNFα^+^IFNγ^-^IL2^-^184 and IFNγ^+^IL2^+^^185^ has been reported to distinguish active from latent pulmonary TB infection. A previous study in Singapore also showed an increased frequency of TNFα^+^CD154^+^IFNγ^+^CD27^-^ and TNFα^+^CD154^+^GM-CSF^+^CD27^-^*Mtb*-specific CD4^+^ T-lymphocytes in active TB as compared to LTBI 184. IL-27, a cytokine that stimulates Th1 differentiation, tend to be higher in tuberculous pleural effusion; whether IL-27 play a significant role in other forms of TB is currently unclear and required further investigation 186. The frequency of CD38^+^HLA-DR^+^Ki67^+^ CD4 T-lymphocytes was found to decrease with ATT, and their detection may thus provide a tool to monitor treatment response and predict treatment success 187.

Previous studies have shown a correlation between ocular inflammation and different immunophenotypes of T-lymphocytes 188-191. Hutchinson *et al.* recently investigated the utility of blood T-lymphocytes specific for *Mtb* antigen (i.e. PPD or ESAT-6 + CFP-10) as an immune surveillance tool to diagnose and manage OTB 192. An increased percentage of PPD-specific CD4^+^ T-lymphocytes from OTB patients (who responded to ATT) expressed CD38 and HLA-DR, which decreased during treatment 192. These CD38 and HLA-DR *Mtb*-specific CD4^+^ T-lymphocytes were additionally identified in peripheral blood, pleural fluid, bronchoalveolar lavage fluid and lung tissue obtained from pulmonary TB patients suggesting a more generalized, non-tissue specific, pathophysiological role of these cells during an *Mtb* infection 193. These studies suggest a potential role for T-lymphocyte profiling in assessing treatment guidance and response and warrants future clinical trials to validate these findings.

## 4. Conclusion

OTB is a serious intraocular disease worldwide with unpredictable treatment outcomes due to an extremely broad clinical spectrum and the lack of reliable, sensitive and specific, yet convenient confirmatory diagnostic tests to help guide the prompt initiation of ATT. Many years of research has introduced many biomarker-based quantitative tools such as IGRA, multiplex PCR and LAMP since the use of the century-old TST. However, distinguishing the underlying pathogenesis of a specific ocular TB phenotype, whether it is due to true *Mtb* infection in ocular structures, a triggered autoimmune response targeting ocular antigens, or a combination of both remains a future objective. Furthermore, more studies are needed to specifically address this potential autoimmune reaction in OTB and its effect on clinical phenotypes and diagnostics. In clinical practice, existing biomarker-based diagnostic approaches continue to be challenged by the issue of cost in low resource settings, lack of immune reaction in concomitant HIV patients, difficulty differentiating latent and active infection and multi-drug resistant *Mtb* infection.

Novel biomarkers ignite hope for a new era of precision-guided molecular diagnosis and individualized treatment approaches. However, precision and personalized medicine are currently limited to genetic approaches without effective integration of individual profiling and disease subtyping at the transcriptome and proteome level 110. The multi-“omics” approach - genomics, transcriptomics, proteomics, and metabolomics, along with cytokines and immunophenotyping through T-lymphocyte profiling has been useful to offer insight into previously unexplainable clinical observations such as strong inflammatory responses despite the paucibacillary nature of OTB and recurrent intraocular inflammation despite prolonged ATT. The immune-mediated inflammatory response suggests more complexity to OTB than simply a precise *Mtb* directed response. Recent studies looking into the complex immunogenesis of OTB postulate that beyond a pathogen-specific immune response triggered by *Mtb* infection, there is a role for autoreactive and mycobacterial specific T-lymphocytes, monocytes/macrophages, natural killer, and dendritic cells as evidenced by the expression of cell-specific chemokines, pro-inflammatory cytokines, and activation markers 135. A broader search for empiric biosignatures is thus needed, which are part of this immunopathogenesis. These markers serves not only to identify the presence of *Mtb* bacilli but also to guide the choice of therapy regimen, monitor progression and treatment response, and predict disease outcome.

While novel multi-omics insights in the *Mtb* directed immune response can provide biomarker signatures beneficial for diagnosis and discrimination between different *Mtb* induced ocular inflammatory processes, these should first be extensively validated in prospective clinical trials and various cohorts. Currently multi-omics approaches remain in the trial phase and the clinical utility of omics-discovered biomarkers in OTB diagnosis and treatment is still to be determined.

There are a number of limitations preventing the widespread use of omics-derived biomarkers. Firstly, most uveitis secondary to autoimmune diseases are also primarily mediated by cellular immunity involving T-lymphocytes. For example, Campisi *et al.* found that apoptosis of *Mtb*-infected host cells enabled generation of self-reactive T_H_17 cells, a subset of helper T-lymphocytes associated with autoimmune diseases 196. Zhong *et al.* established that *Mtb* exposure or genetic susceptibility to TB is a risk factor for uveitis secondary to Behçet disease 197. Moreover, Previously identified pulmonary TB transcriptomic biomarker signatures were upregulated in HIV positive patients and correlated with viral loads in the absence of TB infection 198. Therefore the same molecular profiles or biomarkers of OTB can be presented in the autoimmune and infective conditions and thus may be misleading.

Secondly, as aqueous or vitreous sampling to confirm the presence of *Mtb* are not routinely performed in clinical practice, the lack of a diagnostic gold standard will render the evaluation of predictive parameters (sensitivity, specificity, positive predictive value, negative predictive value) of biomarkers unproductive given the different “gold standards” each independent cohort study compares the same biomarker against. In addition, large heterogeneity in cohort design without comparable output and variation in endemicity may affect the diagnostic potential of identified biomarkers in different populations. Moreover, there are only a limited number of molecular studies available that specifically focus on OTB, each performed on a limited number of clinically presumed OTB patients. The OTB-specific studies mostly comprise HIV-uninfected populations or only minimal numbers of HIV-positive patients. This lowers the power to determine differences and utility of the same biomarker(s) in this sub-group that is prominent in many low-resource, TB-endemic nations. The extent of immunodeficiency from HIV infection also varies widely, hence a large and diverse population of co-infected patients need to be recruited to study the relationship between the accuracy of biomarkers and the extent of immunodeficiency 199. Therefore, multinational centre-based collaborations are essential to increase patient groups, introduce geographic diversity and introduce an international consortium for standardized and feasible “gold standard” diagnostic comparison. The accuracy and utility of biomarkers as diagnostic tools for OTB may improve when integrated with clinical features to generate a composite scoring system informing personalised therapy.

Lastly, though the multi-omics approach is promising, it requires expensive technological and analytic infrastructures which may be unavailable in most settings 200. Some considerations to mitigate the issue of cost include using easily accessible specimens such as tears, and investing in integrated and minimalist platforms with internationally standardized operating procedures.

Moving forward, some current “multi-omics” approaches in pulmonary TB research can potentially be refined and re-aligned for ocular applications. The focus should be on integrating “omics” data from different levels of the biological process and take system-biology approach to find markers that may boost diagnostic accuracy and render a more biologically meaningful understanding of ocular diseases with large heterogeneity. More extensive prospective clinical studies exploring metabolomics in OTB and externally validifying current research in genomics, transcriptomics, proteomics, and T-lymphocyte profiling in OTB beyond the discovery dataset would aid the ultimate development of POC diagnostic tests with high specificity and sensitivity. Eventually, only innovations that are able to translate the empiric findings into large-scale clinical interventions can make a real difference in the burden and outcomes of OTB in high-incidence countries. Therefore, focusing on identifying disease-specific marker panels rather than single marker may help speed up progress in delivering personalized care for patients with OTB.

## Supplementary Material

Supplementary table.

## Figures and Tables

**Table 1:** Studies evaluating the specificity and sensitivity of tuberculin skin test (TST), and three different brands of interferon-gamma release assays (IGRA) - QFT-GIT, QFT-PLUS, T-SPOT.TB.

	Test Name	n	Country	Endemic	Inclusion criteria for Controls	Specificity	Sensitivity
TST							
Fernández-Zamora et al. (2022) 33	TST	191	Brazil	Yes	Diagnosed with uveitis secondary to other non-infectious or non-TB infectious cause; Non-responsive to ATT.	71.8	98.5
Llorenc et al. (2013) 38	TST	103	Spain	No	Diagnosed with uveitis secondary to other non-infectious or non-TB infectious cause; Non-responsive to ATT.	85.7	87.8
Ang et al. (2009) 34	TST	157	Singapore	Yes	Non-responsive to ATT.	72.7	95.5
Cordero-Coma et al. (2010) 42	TST	83	Spain	No	Non-inflammatory eyes.	84	81
Ang et al. (2012) 35	TST	138	Singapore	Yes	Non-responsive to ATT.	51.1	72.0
Ang et al. (2014) 36	TST	106	Singapore	Yes	Bayesian analysis in the absence of gold standard diagnostic.	68.3	70.9
IGRA							
Fernández-Zamora et al. (2022) 33	QFT-GIT, QFT-PLUS	191	Brazil	Yes	Diagnosed with uveitis secondary to other non-infectious or non-TB infectious cause; Non-responsive to ATT.	99.2	90.8
Llorenc et al. (2013) 38	QFT-PLUS	103	Spain	No	Diagnosed with uveitis secondary to other non-infectious or non-TB infectious cause; Non-responsive to ATT.	82.8	90.9
Ang et al. (2009) 34	QFT-GIT	157	Singapore	Yes	Non-responsive to ATT.	81.8	90.9
Cordero-Coma et al. (2010) 42	QFT-GIT	83	Spain	No	Non-inflammatory eyes.	100	81
Babu et al. (2009) 43	QFT-GIT	60	India	Yes	Diagnosed with uveitis secondary to other non-infectious or non-TB infectious cause; Not a close contact of TB patient.	76	82
Ang et al. (2012) 35	T-SPOT.TB	138	Singapore	Yes	Non-responsive to ATT.	75.0	36.0
Ang et al. (2014) 36	QFT-GIT	106	Singapore	Yes	Bayesian analysis in the absence of gold standard diagnostic.	99.6	64.2
Ang et al. (2014) 36	T-SPOT.TB	106	Singapore	Yes	Bayesian analysis in the absence of gold standard diagnostic.	90.6	50.0
Ahn et al. (2014) 37	QFT-GIT	181	Korea	Yes	Non-responsive to ATT.	72.0	100
Gineys et al. (2011) 39	QFT-GIT	96	France	No	Diagnosed with uveitis secondary to other non-infectious or non-TB infectious cause; Non-responsive to ATT.	87.0	84.0

**Table 2:** Studies evaluating the specificity and sensitivity of uniplex PCR with only one gene target, loop mediated amplification assays (LAMP), multiplex PCR with multiple gene (DNA or mRNA-based) targets, GeneXpert Assay, GeneXpert Ultra Assay, MTBDRplus Assay.

	Country (endemicity)	N	Samples	Presumed ocular TB criteria in each study	Targets used	Specificity	Sensitivity	Advantages	Limitations
Uniplex PCR									
Gupta et al. (1998) 79	India (Yes)	10	Aqueous	(a) vasculitis, (b) anterior vitreous cells, (c) snowball, (d) snowbanking, or (e) retinochoroiditis	H_37_RA DNA (150 bp fragment)	33	95	Faster turn-around-time than smear	All NAATs are limited by the inability to differentiate live and dead bacilli, the latter causing false-positivity hence decreasing specificity. Sophisticated and costly electricity-driven equipment required for amplification with limited throughput for PCR, thus it is unsuitable for resource-limited high-endemic regions 64. Variable sensitivity and specificity with different gene targets. IS1081 is a multi-copy gene in the *Mtb* genome, hence increases the yield of detection than other genes that are present as single copy. Up to 40% of North Indian strains of Mtb lack IS6110 gene copy, hence explaining variable sensitivity of PCR in different populations. MPB64 is known to give false-negative results in the concurrent presence of other member(s) mycobacterium family other than *Mtb* (e.g. *M bovis* or *M. fortuitum)* 64.
Arora et al. (1999) 60	India (Yes)	53	Aqueous	Anterior chamber inflammation, with at least one of the following: (a) vasculitis, (b) anterior vitreous cells, (c) snowball, (d) snowbanking, or (e) retinochoroiditis	H_37_RA DNA (150 bp fragment)	95.3	37.7		
Gupta et al. (2003) 80	India (Yes)	5	Aqueous or vitreous	Serpiginous choroiditis	IS6110	33	89		
Singh et al. (2012) 81	India (Yes)	11	Vitreous	Eales disease	MPB64	33	95		
Murugan et al. (2016) 61	India (Yes)	22	Aqueous or vitreous	Clinical history comprehensive ophthalmic examination, systemic and ocular investigations	MPB64	80	42		
Sudheer et al. 2018 62	India (Yes)	56	Aqueous and/or vitreous	Clinical features: hypopyon, granulomatous keraticpercipitate, iris, choroid, or disc granulomas, active vasculitis, choroiditis, and healed chorioretinal scars along blood vessels; minimum 6 months of follow-up; no response to oral steroids	MPB64	92.3	73.3		
Sharma et al. (2019) 63	India (Yes)	200	India (Yes)	(2) all known causes of infectious uveitis except TB and known noninfectious uveitic syndromes ruled out; (3) positive tuberculin skin test (4) received antitubercular therapy for a minimum of 12 months (6) no recurrence of uveitis. Out of these 70 cases, 3 were culture-positive and 67 were culture negative for *M. tuberculosis*.	MPB64	100	68.2		
Sharma et al. (2019) 63	India (Yes)	200	India (Yes)		IS6110	100	66.4		
Sharma et al. (2020) 64	India (Yes)	120	Vitreous		IS1081	100	61.42		
LAMP									
Balne et al. (2013) 68	India (Yes)	14	Aqueous or vitreous	Criteria as per described in 19	MPB64	100	85.7	Easily administered in the rural setting through battery-operated water baths, simple instructions requiring minimal staff training, and rapid results within one hour. 64 Cost less than 1 USD per sample. 64 High specificity. Higher sensitivity than PCR using the same target gene(s) as LAMP uses three primer pairs to recognize more regions of the target gene(s). 64	Specificity is still limited, although more than four-fold that of GeneXpert. Insufficient external validation with different geographical populations harboring different genotypes of the *Mtb* complex (MTBC) to be WHO-endorsed 64. Unable to detect rifampicin or isoniazid resistance.
Sharma et al. (2020) 64	India (Yes)	120	Vitreous	(2) all known causes of infectious uveitis except TB and known noninfectious uveitic syndromes ruled out; (3) positive tuberculin skin test (4) received antitubercular therapy for a minimum of 12 months (6) no recurrence of uveitis. Out of these 70 cases, 3 were culture-positive and 67 were culture negative for *M. tuberculosis*.	MPB64	100	67.14		
Sharma et al. (2015) 82	India (Yes)	30	Vitreous and 1 iris biopsy	Confirmed by positive multitargeted PCR for *M tuberculosis* from intraocular samples	IS6110	100	70		
Sharma et al. (2020) 64	India (Yes)	120	Vitreous	(2) all known causes of infectious uveitis except TB and known noninfectious uveitic syndromes ruled out; (3) positive tuberculin skin test (4) received antitubercular therapy for a minimum of 12 months (6) no recurrence of uveitis. Out of these 70 cases, 3 were culture-positive and 67 were culture negative for *M. tuberculosis*.	IS6110	100	64.28		
Sharma et al. (2020) 64					IS6110, MPB64, IS1081	100	77.14		
Sharma et al. (2020) 64					IS1081	100	71.42		
DNA-based Multiplex PCR									
Biswas et al. (2016) 83	India (Yes)	21	Aqueous	MSC or choroiditis suspected for TB	IS6110, MPB64	50	98	Combination of multiple gene targets has better yield of detection (sensitivity) than compared to a single gene target used. Rapid results within2-3 hours. 64	Same limitations as Uniplex PCR. Costs nearly 10 times that of LAMP 64. In settings endemic for MDR-TB, MPCR is unable to detect rifampicin or isoniazid resistance, hence an additional step of gene sequencing to search for resistance genes will cost approximately 20 USD with a turn-around time of 2-3 days 63. Specificity is still limited, although more than four-fold that of GeneXpert, and more than two-fold that of MTBDR Assay.
Mohan et al. (2014) 84	India (Yes)	13	Aqueous	MSC or choroiditis suspected for TB	IS6110, MPB64, and protein b	60	62		
Agarwal et al. (2019) 47	Multiple countries (most samples from India	49	Aqueous and/or vitreous	Clinical signs suggestive of uveitis TB and others where the specific cause had been excluded; corroborative evidence suggestive of uveitis TB	IS6110, MPB64, and protein b	80	93		
Sharma et al. (2013) 65	India (Yes)	9	Aqueous or vitreous	Clinical signs suggestive of uveitis TB with other specific causes excluded; corroborative evidence suggestive of uveitis TB	IS6110, MPB64, and protein b	100	77.77		
Sharma et al. (2019) 63	India (Yes)	200	vitreous	(2) all known causes of infectious uveitis except TB and known noninfectious uveitic syndromes ruled out; (3) positive tuberculin skin test (4) received antitubercular therapy for a minimum of 12 months (6) no recurrence of uveitis. Out of these 70 cases, 3 were culture-positive and 67 were culture negative for *M. tuberculosis*.	IS6110, MPB64 and protein b	100	71.8		
Sharma et al. (2022) 66	India (Yes)	75	vitreous		IS6110, MPB64 and protein b	100	72		
Sharma et al. (2022b) 67	India (Yes)	39	vitreous	Signs of active uveitis or A positive tuberculin skin test as per the Center of disease control (CDC) guidelines, or chest X-ray suggestive of TB was present. All other known causes of infectious or non-infectious uveitis were excluded.	IS6110, MPB64 and protein b	100	73.7		
mRNA-based Multiplex PCR									
Sharma et al. (2022b) 67	India (Yes)	39	vitreous	Signs of active uveitis or A positive tuberculin skin test as per the Center of disease control (CDC) guidelines, or chest X-ray suggestive of TB was present. All other known causes of infectious or non-infectious uveitis were excluded.	IS6110, MPB64 and protein b	100	42.1	Able to detect “viable” *Mtb* bacilli from dead bacilli as the half-life of bacterial mRNA is extremely short (∼9.5 minutes *in vitro*), hence better reflecting mycobacterial viability and potentially useful for monitoring susceptibility to ATT 67	Degradation of mRNA during storage and extraction procedures renders poorer sensitivity than DNA-based MPCR, thus may not be sufficient to rule out OTB.
GeneXpert Assay									
Sharma et al. (2019) 63	India (Yes)	200	vitreous	(2) all known causes of infectious uveitis except TB and known noninfectious uveitic syndromes ruled out; (3) positive tuberculin skin test (4) received antitubercular therapy for a minimum of 12 months (6) no recurrence of uveitis. Out of these 70 cases, 3 were culture-positive and 67 were culture negative for *M. tuberculosis*.	IS6110, rpoB	100	17.2	Rapid results within2-3 hours. 63 Simple cartridge-based real-time PCR eliminated the problem of cross-contamination because of self-contained cartridges. 64 This confers low biosafety risk and staff require minimal training. 63 Detects rifampicin resistance.	Costs 15 USD per sample 63. Poor sensitivity due to lower analytical sensitivity of Xpert (131 CFU/ml in spiked sputum) in comparison to 2-3 CFU/ml for MPCR 63. Specimen may be diluted when reagent is added for DNA extraction. Sensitivity is too low to serve as a reliable test for ruling out OTB. False-positive rifampicin resistance due to silent mutations that probe-based molecular tests like Xpert cannot differentiate from true-positive resistance 63. Does not detect isoniazid resistance.
Sharma et al. (2022) 66	India (Yes)	75	vitreous		IS6110, rpoB	100	16		
Sharma et al. (2022b) 67	India (Yes)	39	vitreous	Signs of active uveitis or A positive tuberculin skin test as per the Center of disease control (CDC) guidelines, or chest X-ray suggestive of TB was present. All other known causes of infectious or non-infectious uveitis were excluded.	IS6110, rpoB	100	10.5		
GeneXpert Ultra Assay									
Sharma et al. (2022) 66	India (Yes)	75	vitreous	(2) all known causes of infectious uveitis except TB and known noninfectious uveitic syndromes ruled out; (3) positive tuberculin skin test (4) received antitubercular therapy for a minimum of 12 months (6) no recurrence of uveitis. Out of these 70 cases, 3 were culture-positive and 67 were culture negative for *M. tuberculosis*.	IS108, IS6110, rpoB	100	50	Circumvents the identification of silent mutations as rpoB gene mutations by using high resolution melt (HRM) curve analysis rather than probe-based chemistry. 66	Same limitations as GeneXpert.
MTBDRplus Assay									
Sharma et al. (2019) 63	India (Yes)	200	vitreous	(2) all known causes of infectious uveitis except TB and known noninfectious uveitic syndromes ruled out; (3) positive tuberculin skin test (4) received antitubercular therapy for a minimum of 12 months (6) no recurrence of uveitis. Out of these 70 cases, 3 were culture-positive and 67 were culture negative for *M. tuberculosis*.	rpoB, katG and inhA	100	34.5	Able to establish diagnosis of MDR-TB by detecting rifampicin and isoniazid resistance, especially in areas endemic for isolated rifampicin resistance. Separate working stations required, hence reducing risk of cross- contamination. 63	Poor sensitivity due to lower analytical sensitivity of MTBDRplus assay (160 CFU/ml in spiked sputum) in comparison to 2-3 CFU/ml for MPCR 63. Sensitivity is too low to serve as a reliable test for ruling out OTB. False-positive rifampicin resistance due to silent mutations that probe-based molecular tests like MTBDR Assay cannot differentiate from true-positive resistance 63. Space-consuming requiring designated areas for a proper setup 63. Time-consuming as turn-around time is 24 hours 63. More expensive than LAMP, MPCR and GeneXpert costs 22 USD per isolate 63. Limited accessibility and throughput in endemic resource-limited regions 66.

**Table 3:** Selection criteria of sample and control groups in each novel OTB biomarker study, advantages and disadvantages of each -omics method.

	Author (Year)	Biomarkers discovered	Specimen	Subject (n)	Control (n)	Advantages	Disadvantages
Transcriptomic	Chandalawada et al. (2022) 103	Four miRNAs, miR-423-5p, miR-328-3p, miR-21-5p, and miR-16-5p, were significantly dysregulated in aqueous humor of OTB patients	Aqueous humor	Confirmed OTB as defined with positive TB PCR test (n = 5). Presumed OTB is with another positive result of follow-up examinations or positive ATT response (n = 2).	Patients with only cataract (n = 2).	Generally speaking, the nucleic acid-based omics approaches for data generation rely on five major steps: appropriate sample collection, high-quality nucleic acid extraction, library preparation, clonal amplification, and sequencing. For the last step, sequencing-based technologies, the most advanced of the omics technologies in terms of availability of laboratory reagents for standardized protocols, analytical tools and public databases for data sharing, provide unique opportunities to obtain high quality from small amounts of tissues or individual cells to address a wide range of biological questions 194. Peripheral blood samples are easy to obtain.	Micro-RNA to date lack external validation and their findings cannot be compared due to relevant methodological differences in processes such as RNA extraction or data analysis 102. Heterogeneous datasets pose challenges because quality assurance, quality control, data normalization and data reduction methods differ among the various types of individual datasets 195. Small sample size for OTB. Larger validation studies are needed to externally validify discovery dataset.
	La Distia Nora et al. (2018) 120	IFN signature based on 10 interferon-stimulated genes (UBE2L6, FCGR1B, GBP1, IL1B, MYD88, TLR8, IRF7, STAT1, SERPING1, and IFIT2) could discriminate between active pulmonary TB and healthy controls with a sensitivity of 100% and a specificity of 91%.	Peripheral blood	Active Pulmonary TB Without uveitis, Uveitis with clinically diagnosed active pulmonary TB, QFT (+) uveitis of unknown cause. (n = 80) Mtb sputum-positive active pulmonary TB patients (HIV negative) without uveitis or a history of ATT as a positive control group. (n = 10)	QFT-negative, had no history of uveitis and did not use any medication at the time of the study. (n = 23)		
	Schrijver et al. 121	10-gene type 1 IFN signature (UBE2L6, FCGR1B, GBP1, IL1B, MYD88, TLR8, IRF7, STAT1, SERPING1, and IFIT2) displayed an inverse correlation with serum complement component C1q.	Peripheral blood	APTB uveitis unknown (n = 50) QFT- Uveitis (n = 51) QFT+ Uveitis of unknown aetiology (n = 58) APTB‐assoc. uveitis (n = 12) APTB w/o uveitis (n = 10)	Healthy controls (n = 73) Primary Sjögren's syndrome (n = 86) Systemic lupus erythematosus (n = 30) Systemic sclerosis (n = 23)		
Proteomic	Ang et al. (2012) 134	Inflammatory cytokines such as IL-6 and CXCL8/IL-8 and Th1 associated chemokines CXCL9, and CXCL10 were significantly increased in the TB-associated uveitis group compared to the non- inflammatory controls, and it is also distinct from the cytokine profiles of idiopathic uveitis.	Aqueous humour	TB-associated uveitis by presenting with acute, active uveitis, with clinical signs of granulomatous inflammation, broad-based posterior synechiae, or retinal vasculitis, with or without choroiditis, and positive QFT or T-SPOT.TB, and TST indurations ≥15 mm, and respond to ATT. (n = 10) Idiopathic uveitis with no evidence of TB (negative TST (TST<10 mm) and negative IGRA) or other diseases. (n = 13)	Patients with no ocular pathology other than cataract were enrolled as non-inflammatory controls. (n =23)	The development of improved methods in quantitative proteomics (Mass Spectrometry‐based techniques) has increased the relevance of proteins that complements both genomics and traditional biochemical techniques to aid better understanding of the complex interaction between *Mtb* and host 5. Recent advances in instrument sensitivity while decreasing the amount of sample required for high-throughput analyses and now allow for the detection of minimal differences in protein abundances 194. The development of effective isotopic labeling tools for tissue samples have significantly improved the accuracy and reproducibility of peptide and protein quantification using MS 194.	There is no consensus in terms of data formatting, cleaning and normalization 194. Proteomics approaches still require significant amounts of sample due to the lack of protein amplification methods, and face difficulties in isolation of membrane proteins, detection of low abundance proteins and insoluble proteins. The reliance on separation of complex chemistries (i.e., different charged states and post-translational modifications) using chromatography adds to variability in protein quantification in top-down and bottom-up proteomics 194. There is variability in peptide identification due to variation in peptide structure, charge and hydrophobicity, and these biochemical properties of peptides and proteins affect their ability to be detected and identified by NMR or MS. Analysis pipelines for proteomic data must deal with absent data (i.e., is the peptide not detected because it is not ionized efficiently, or is it truly not present in the sample), normalization and absolute versus relative quantification 194.
	De Simone et al. (2022).^135^	Lower concentrations CXCL13, CXCL-8, CXCL-10 in AH samples for TBU and Q +OS groups (with no significant difference between groups) than definite OS group. The three chemokines were elevated in AH samples than in peripheral blood, suggesting an intraocular production and supporting their possible role as therapeutic targets.	Aqueous humour, Peripheral blood	Presumptive TBU was based on the positivity of the Q-Gold test and compatible clinical ocular features accompanied by a negative workup for other causes of uveitis. (n = 12)	Controls underwent phacoemulsification intervention for cataract and cornea surgery and who were not affected by any other concomitant inflammatory and/or infectious disease nor had a prior history of uveitis. (n = 9) Definite OS based on the histopathological identification of non-necrotizing epithelioid cell granulomas, negative AFB and clinically compatible ocular signs. (n = 15) Definite OS associated with Q-Gold positivity (Q + OS). (n = 5)		
	Abu El-Asrar et al. (2012) 138	Elevated CXCL8 and CXCL10 levels in aqueous humour samples of presumed OTB patients.	Aqueous humour	Presumed TBU if consistent ocular findings, no other cause of their uveitis, positive TST (⩾15mm), response to ATT. (n = 14)	Cataract extraction with no prior history of uveitis. (n = 30)		
	Schrijver et al. (2022) 139	Vitreous CCL17 and CXCL13 levels were found to distinguish sarcoid uveitis from TB‐associated uveitis (significantly lower), with a sensitivity of 67% and a specificity of 78%.	Vitreous humour	Cohort 1: TB‐associated uveitis (n = 6). Cohort 2 (blinded): TB‐associated uveitis (n = 0).	Cohort 1: sarcoid uveitis (n = 15), (P)VRL (n = 7), TB‐associated uveitis (n = 6), non‐TB infectious uveitis (n = 6), uveitis associated with systemic disease (n = 2), idiopathic uveitis (n = 8), masquerade syndrome other than (P)VRL (n = 3).		
	Bansal et al. (2021) 141	Vitreous protein analysis found that OTB patients showed 11 upregulated differentially expressed proteins (DEPs) and 21 downregulated DEPs compared to a non-TB uveitis or non-uveitis patients.	Vitreous humour	Presumed TBU, which was diagnosed by the presence of active uveitis with characteristic clinical ocular signs, and supported by corroborative evidence of TST ≥ 10 mm of induration at 48-72 h and/or a positive X ray/CT scan of chest. (n = 3) Confirmed TBU with triplex PCR. (n = 10)	Positive control groups consisted of with active uveitis with clinical signs suggestive of causes other than TBU. All these samples also had a negative triplex PCR for MTB. (n = 7) Negative control group had no evidence of any intraocular inflammation, and underwent vitreous surgery for various vitreoretinal disorders such as macular hole, epiretinal membrane, dropped nucleus, etc. (n = 9)		
	Van der Colff et al. (2023) 142	29 biomarkers were tested on both serum and urine samples. Most biomarker concentrations were significantly higher in serum than in urine (p < 0.01). Only 2 (IL-1RA and IL-2) showed higher concentrations in urine than serum (p < 0.01). Three biomarkers (sIL-2Ra, sTNFRI and IFNγ) showed no difference in concentration between urine and serum (p > 0.05).	Peripheral blood sample and urine sample.	Most patients were diagnosed with possible OTB and only one with confirmed OTB (n = 14)	No control (n = 0)		
Stimulation Assay	Makhoba et al. (2021) 167	Four-marker biosignature comprising of CD40 ligand, IL-33, IFN-γ, and SAP, which showed potential in diagnosing OTB.	Peripheral blood	OTB diagnosed when other causes of ocular inflammation excluded, at least one suggestive clinical sign as well as evidence of TB on CXR or elsewhere in the body. (n = 32)	Other intra-ocular diseases (n = 60)	Peripheral blood samples are easy to obtain. *In vitro* stimulation is safe for the patient; no risk of triggering infectious or allergic reaction. Potentially able to differentiate active and latent OTB by detecting the difference in cytokine levels in both states.	Small sample size for OTB. Larger validation studies are needed to externally validify discovery dataset. High TB burden setting may misdiagnose latently infected OTB with active OTB. As latently infected individuals will habour T cells which will recognize the antigens used in QFT tubes, and also secrete host markers into QFT supernatants irrespective of the primary ocular diagnosis. T cell responses may not be limited to the use of single peptides for eliciting both the antimycobacterial and retinal antigen-specific responses. T cell responses to other immunodominant peptides (non-mycobacterial and non-retinal) may confound biomarker discovery specific to immune response triggered by OTB. Does not provide the phenotype of immune cells producing biomarkers. Obtaining vitreous humour via pars plana vitrectomy is invasive.
	Alam et al. (2022) 168	TST-positive undifferentiated uveitis (UNK) generates a stronger monofunctional and polyfunctional (dual-cytokine) intraocular cytokine response than active OTB.	Vitreous humour	Patients who fulfilled the SUN classification criteria for OTB (retinal vaculitis, SLC, MFC, intermediate or panuveitis with positive TST and negative tests for sarcoidosis and syphilis) and/or tested positive for TB-PCR. (n = 23)	Patients with a positive TST who did not fulfill the SUN criteria and who had a negative TB-PCR study, classified as uveitis of unknown origin (UNK). (n = 24) TST-negative patients, with or without a well-defined non-TB uveitis entity were classified as non-TB control subjects. (n = 24)		
Serum and intraocular protein analysis	Singh et al. (2021) 177	Raised VEGF and decreased FGF levels in RPE cells and vitreous humour, but not in tears.	Vitreous humour and tears.	IS6110 PCR-positive vitreous samples were considered confirmed OTB. (n = 15) Tears collected from clinically suspected OTB patients. (n = 15)	Clinically non-OTB uveitis and IS6110 PCR-negative vitreous samples. (n = 27) Tears collected from clinically non- OTB patients. (n = 20)	Potential to improve clarity on pathogenesis of OTB presentation (e.g. retinal vasculitis) and may spur research for other therapeutic agents (e.g. anti-VEGF).	Small sample size. Serial dilution of vitreous samples may cause variability in bacteria density. Tears, an easily obtainable sample, unfortunately did not show similar changes in VEGF and FGF levels as in RPE cells and vitreous humour. Obtaining vitreous humour via pars plana vitrectomy is invasive. Unable to confirm diagnosis of OTB; still relies on presumable diagnosis of OTB.
T-lymphocyte profiling	Hutchinson et al. (2021) 192	Only increased CD38 and HLA-DR expression on Mtb-specific CD4 T cells were significant to discriminate different OTB phenotypes and predict treatment response.	Peripheral blood	At least one of the three tests (chest radiography, Mantoux test, IGRA) positive. Two more patients were diagnosed with OTB, 1 likely had diagnosis of TB uveitis due to presence of clinical finding with panuveitis and occlusive vasculitis in a patient from area endemic with TB (Bangladesh) while the other 1 had previously known history of positive Mantoux test and T-SPOT.TB. (n = 36)	Not Applicable (n = 0)	Provides the phenotype of immune cells as well as their change in cytokine expression (upregulation or downregulation). Potential to discriminate treatment responders from non-responders. In other words, this can differentiate those with active OTB who will benefit from ATT versus those with latent ATT that can experience harmful side effects from overly-aggressive treatment. Peripheral blood samples are easy to obtain.	Small sample size. Unable to confirm diagnosis of OTB; still relies on presumable diagnosis of OTB.

## Abbreviations

IS6110: Insertion sequence 6110
rpoB: Ribonucleic Acid Polymerase Beta Subunit
katG: Tuberculosis catalase-peroxidase enzyme
inhA: Inhibin Subunit Alpha
C1q: complement component 1q
CD4+: Cluster of differentiation 4
CD27: Cluster of differentiation negative 27
CD38+: Cluster of differentiation 38
CD40L: Cluster of differentiation 40 Ligand
CD154+: Cluster of differentiation 154
IL-1β: Interleukin 1 beta
IL-2: Interleukin 2
IL-6: Interleukin 6
IL-8: Interleukin 8
IL-10: Interleukin 10
IL-15: Interleukin 15
IL-17: Interleukin 17
IL-22: Interleukin 22
IL-33: Interleukin 33
CXCL8: chemokine (C-X-C motif) ligand 8
CXCL9: chemokine (C-X-C motif) ligand 9
CXCL10: chemokine (C-X-C motif) ligand 10
CXCL13: chemokine (C-X-C motif) ligand 13
TNF-α: Tumour necrosis factor-alpha
TGF-β: Transforming growth factor beta
TB7.7: Tuberculosis 7.7 antigen
CCL4: Chemokine (C-C motif) ligand 4
CCL8: Chemokine (C-C motif) ligand 8
CCL17: Chemokine (C-C motif) ligand 17
MIP-1β: Macrophage inflammatory protein-1 beta
MIP-3α: Macrophage inflammatory protein-3 alpha
KRAS: Kirsten Rat Sarcoma viral oncogene homolog
MTORC1: Mechanistic target of rapamycin complex 1
MCP2: monocyte chemoattractant protein 2
SAP: Serum Amyloid P-Component
HLA-DR+: Human Leukocyte Antigen - DR isotype
GM-CSF+: Granulocyte-macrophage colony-stimulating factor
