# Supplementary material for: Diagnosis and biomarkers for ocular tuberculosis: From the present into the future
Source: Theranostics. 2023 Apr 1;13(7):2088–113. doi: 10.7150/thno.81488 (PMC10157737; doi:10.7150/thno.81488)
Supplement: Supplementary file 1 — Supplementary table. [file thnov13p2088s1.pdf]

Supplementary Table: Advantages and Disadvantages of individual OTB novel biomarkers.

	Author (Year)	Biomarkers Discovered	Technique	Advantages	Disadvantages
Transcriptomic	Chandalawada et al. (2022)	Four miRNAs, miR-423-5p, miR-328-3p, miR-21-5p, and miR-16-5p, were significantly dysregulated in aqueous humorofOTB patients.	Small-RNA sequencing using Real-time quantitative PCR (RT-qPCR) and next-generation sequencing (NGS).	Internal validation performed using 2 OTB samples and 2 cataract controls.<br><br>miRNA are small, stable RNAs that play an important role in regulating target gene expression to activate macrophages in response to *Mtb*.<br><br>Of the four dysregulated miRNAs identified, miR- 423-5p, miR-21-5p and miR-16-5p are altered in systemic TB, while miR-328-3p is not reported in TB elsewhere. All four miRNAs play a role in IOTB pathogenesis via PI3K-Akt signaling, MAPK pathway, Autophagy and tuberculosis pathway, making these altered miRNAs future candidates for distinguishing active OTB from latent TB and health individuals, as well as potentially help evaluate treatment response.	No further external validation study was performed after this discovery data set.<br><br>Low-input total RNA of each sample used for small-RNA sequencing, which would possibly affect the fold change. For instance, miR-16-5p was not concordant with two technologies.<br><br>Small sample size in the NGS might bias the low abundant miRNAs expression during normalization,
	La Distia Nora et al. (2018)	IFN signature based on 10 interferon-stimulated genes (UBE2L6, FCGR1B, GBP1, IL1B, MYD88, TLR8, IRF7, STAT1, SERPING1, and IFIT2)<br>could discriminate between active pulmonary TB and healthy controls with a sensitivity of 100% and a specificity of 91%.	RT-qPCR	Elevated expression of type 1 IFN-inducible gene transcripts without concurrent elevated MxA expression that is common in systemic autoimmune diseases, including systemic lupus erythematosus (SLE) strongly supports type 1 IFN activity to be of active TB origin.<br><br>Ten type 1 IFN-inducible gene transcripts were identified as a 10-gene biosignature. When a type 1 IFN signature score was applied to this 10-gene set, a score ≥5.61 displayed the optimal sensitivity (100%) and specificity (91%) for distinguishing active pulmonary (*Mtb* sputum smear-positive) TB patients without uveitis from healthy controls. In line with this result, the two TB uveitis cases diagnosed with active pulmonary TB and having positive *Mtb* sputum smear displayed a type 1 IFN signature score >5.61. This finding indicates that microbiologically proven active pulmonary TB with or without uveitis is associated with high expression of type 1 IFN-inducible genes. However, several additional uveitis cases, who were QFT-positive, displayed a positive type 1 IFN signature (≥5.61) despite being *Mtb* sputum negative, which is predicted to have a higher likelihood of having uveitis secondary to OTB and beneficial reaction to ATT.	Lacks the results of type 1 IFN signature in QFT-positive patients without uveitis.<br><br>QFT-negative uveitis patients were also excluded as they are never suspected of TB-associated uveitis, hence it is unconfirmed if a proportion of QFT-negative uveitis cases will be associated with a positive type-1 IFN gene signature, for instance in case of uveitis in association with autoimmune disease or toxoplasmosis.<br><br>External validation by **[Schrijver *et al.*]** contradicts the hypothesis. The latter study found that active TB can exist in the context of a negative type-1 IFN signature, in the presence of elevated serum C1q levels.

	Schrijver et al. (2020)	10-gene type 1 IFN signature (UBE2L6, FCGR1B, GBP1, IL1B, MYD88, TLR8, IRF7, STAT1, SERPING1, and IFIT2) displayed an inverse correlation with serum complement component C1q.	RT-qPCR, ELISA	Two independently collected cohorts of Indonesian patients with APTB and healthy controls were assessed, allowing for internal validation.<br><br>Serum C1q levels displayed a significant inverse correlation with the type-1 IFN signature scores in active pulmonary TB (APTB) patients, while serum C1q did not show any correlation with the peripheral blood type-1 IFN signature scores in any of these systemic autoimmune diseases.<br><br>C1q levels were significantly elevated in serum from QFT$^+$ patients with uveitis of unknown aetiology as compared to QFT$^-$ uveitis patients and healthy controls. Serum C1q levels were comparable between QFT$^-$ uveitis patients and healthy controls. Comparison between infectious and non-infectious uveitis within the QFT$^-$ uveitis group revealed no difference in serum C1q levels. Interestingly, APTB patients with uveitis displayed significantly higher serum C1q levels than APTB patients without uveitis. This shows that elevated C1q levels are rather specific to QFT$^+$ patients with uveitis and APTB patients with uveitis. The combined measurement of serum C1q and type-1 IFN signature score yielded a sensitivity of 100% and specificity of 87%, thus outperforming the diagnostic accuracy of serum C1q or type-1 IFN signature score alone.	Not all included healthy controls were examined for the presence of LTBI, which could have served as a separate control group.<br><br>Disease-specific gene signatures were used for TB cohort and systemic autoimmune disease cohort, hence the possibility of both TB and autoimmune diseases having the same correlation between serum C1q and the same type 1 IFN biosignature set (if this was used for both cohorts) cannot be excluded.<br><br>Contradicts previous report that QFT$^+$ uveitis patients with a negative type-1 IFN signature is sufficient to stratify as low risk for OTB. **[La distia Nora et al.]** Current data indicate that active TB can exist in the context of a negative type-1 IFN signature, in the presence of elevated serum C1q levels.<br><br>No data on outcome/treatment response available to support the hypothesis that those stratified as high risk for OTB will benefit from ATT.<br><br>It remains unknown how C1q's role in regulating type-1 IFN signalling during active TB disease relates to disease pathology, duration, activity or progression.
Proteomic	Ang et al. (2012)	Inflammatory cytokines such as IL-6 and CXCL8/IL-8 and Th1 associated chemokines CXCL9, and CXCL10 were significantly increased in the TB-associated uveitis group compared to the non-inflammatory controls, and it is also distinct from the cytokine profiles of idiopathic uveitis.	Fluorescence intensity (FI) from Magnetic color-bead-based multiplex assay	Abnormal concentrations of various cytokines in the aqueous humour can potentially provide an alternative for diagnosing conditions that may otherwise be considered as idiopathic in patients with uveitis.<br><br>Step-wise analysis and mathematical modeling with decision tree analysis were used to account for the complicated intrinsic interactions between cytokines.<br><br>By deleting highly correlated cytokines and cytokines that were not significantly different between groups, the analysis revealed a panel of cytokines that distinguishes samples with TB-associated uveitis from non-inflammatory controls.	Small number of subjects and the cross-sectional design, in which all subjects had aqueous sampling and analysis during active disease but not post-ATT.<br><br>Lack of aqueous sample control from eyes of healthy individuals with no ocular pathology to serve as controls for future studies.<br><br>Random recruitment subjects with uveitis and non-inflammatory controls led to a significant difference in the mean age between groups, although these differences did not show up as significant changes during multivariate analysis.

	De Simone et al. (2022)	Lower concentrations CXCL13, CXCL-8, CXCL-10 in AH samples for TBU and Q +OS groups (with no significant difference between groups) than definite OS group. The three chemokines were elevated in AH samples than in peripheral blood, suggesting an intraocular production and supporting their possible role as therapeutic targets.	Flow cytometry	First study that simultaneously analyse and compared cytokine levels in AH samples of patients with definite OS, presumptive TBU and overlapping granulomatous uveitis (Q + OS).<br><br>Elevated CXCL-10 levels in OS and elevated CXCL-8 levels in TBU were concordant with previous research, while the comparison between the degree of elevation between the two groups were compared in this study.	Low number of patients with different anterior chamber cells included in the three groups.
	Abu El-Asrar et al. (2012)	Elevated CXCL8 and CXCL10 levels in aqueous humour samples of presumed OTB patients.	Cytokine and chemokine assays which is a cocktail of antibody-coated non-magnetic beads.<br><br>Cytokines: IL-4, IL-10, IL-12, IL-15, IL-17, IFN-γ and TNF-α. Chemokines: IL-8, GRO-α, MIG, IP-10 and SDF-1	Increased levels of the proinflammatory cytokines IL-15, IL-17, IFN-γ, and TNF-α and the immunosuppressive cytokine IL-10. On the other hand, IL-4 and IL-12 were not detected. The neutrophil chemoattractants GRO-α and IL-8, and the lymphocyte chemoattractants MIG and IP-10 levels were elevated. In contrast, no significant difference was observed in SDF-1 levels between patients and controls. It is interesting to note that for the CXCR2 ligands, the mean levels of GRO-α were 6-fold higher than those of IL-8, and that for the CXCR3 ligands, the mean levels of IP-10 were 15-fold higher than those of MIG. Collectively, these findings suggest that the cytokine status within the AH from patients with PTU was polarized toward a Th_1 response (IFN-γ levels were 23-fold increased, and IP-10 levels were 190-fold enhanced), whereas Th_2 cytokine responses are not enhanced and that both Th_1 and Th_{17} subsets are involved in the immunopathogenesis of PTU. The cytokine status was polarized strongly toward a Th_1 response. These findings also suggest a role of specific CXCR2 and CXCR3 ligands in the chemoattraction of neutrophils and activated lymphocytes, respectively, in patients with PTU.<br><br>These findings are consistent with previous observations that Th_2 responses are not enhanced in human tuberculosis. Serum levels of the Th_2 cytokine IL-4 were not elevated in patients with tuberculosis.	
	Schrijver et al. (2022)	Vitreous CCL17 and CXCL13 levels were found to	Olink proximity	Comparison between sarcoid uveitis and TB-associated uveitis revealed significantly lower levels of CCL17	However, in the validation data set: the average CCL17 concentration did not differ significantly between

		distinguish sarcoid uveitis from TB-associated uveitis (significantly lower), with a sensitivity of 67% and a specificity of 78%.	extension immunoassay, followed by Luminex magnetic bead-based assays to measure (CCL)2, CCL17, CD40, (CXCL)13, FASL, IL-6 and IL-10. Ezrin (EZR) was measured by ELISA for validation.	and CXCL13 in TB-associated uveitis.<br><br>Most likely, this finding was due to decreased CCL17 vitreous levels in TB-associated uveitis, as these were significantly lower than other uveitis cases (sarcoid uveitis excluded).<br><br>Vitreous CCL17 levels were found to distinguish sarcoid uveitis from TB-associated uveitis, with a sensitivity of 67% and a specificity of 78%.<br><br>This finding could improve stratification of QFT-positive uveitis cases without further clinical signs of TB.	vitreous of sarcoid uveitis and TB-associated uveitis; immunoassay displayed measurable CCL17 vitreous levels in 50% of the histologically confirmed sarcoid uveitis patients, while none of the vitreous from TB-associated uveitis patients contained measurable levels.<br><br>Retrospective validation of the classifier in the undiagnosed cases presenting with uveitis-like symptoms in cohort 2 was limited by the sample size, which only contained two patients with sarcoid uveitis and no patients with TB-associated uveitis. TB-associated uveitis was not sufficiently represented to validate the findings in cohort 1. Further thorough validation in larger cohort studies is needed.<br><br>Additionally, identification of false-positive biomarkers or overestimation of biomarker importance can more readily occur with limited sample size.<br><br>Furthermore, due to the lack of long-term follow-up data of included patients, we cannot formally exclude the possibility of diagnostic misclassification of the idiopathic patients, or of a future (P)VRL diagnosis, which would then affect the calculated sensitivities and specificities in our present study.
	Bansal et al. (2021)	Vitreous protein analysis found that OTB patients showed 11 upregulated differentially expressed proteins (DEPs) and 21 downregulated DEPs compared to a non-TB uveitis or non-uveitis patients.	C-18 reversed-phase LC-MS/MS.	Insulin-like growth factor 2 messenger RNA binding protein 3 (IGF2BP3) as the most significantly upregulated protein in TBU group compared to both the controls. IGF-I is known to prime the immunocompetent cells to respond to infection.<br><br>The TBU (group A) proteins revealed an upregulation of coagulation cascades, complement and classic pathways, and downregulation of metabolism of carbohydrates, gluconeogenesis, glucose metabolism and glycolysis/gluconeogenesis pathways as compared to positive and negative controls (groups B + C combined). The complex interplay between the coagulation system and host inflammatory response in TB suggests the role of coagulation cascade in generation of fibrin, leading to granuloma formation in TB<br><br>As per the sub-group analysis of comparing TBU	The sample size is small, and shows variations among the number of samples in different groups (Group A = 13 samples, Group B = 7 samples and Group C = 9 samples). As a result, the bias introduced by confounders such as severity of disease and treatment outcome cannot be adjusted.<br><br>Collection of ‘diluted’ vitreous sample is a preferred practice while doing PPV to ensure safety of the eye during the surgical procedure. This further limits research on intraocular fluids, when compared with ‘undiluted’ vitreous. Also, the DEPs need validation. Further, our samples were lyophilised for the ease of transport. An undesirable protein degradation (denaturation, aggregation, decreased potency, etc.) may be inevitable, resulting in alteration of protein structure.<br><br>The presence of cytokeratins as ubiquitous

				(Group A) with non-TBU (Group B), Keratin, type I cytoskeletal 14 (KRT14) was the most upregulated protein, followed by Beta-crystallin B2 (CRYBB2), Prolactin-inducible protein (PIP), Dystroglycan (DAG1) and Dermcidin (DCD).<br><br>The upregulation of apoptosis, KRAS signaling, diabetes pathways, classic pathways, etc, and downregulation of MTORC1 signaling, glycolysis/gluconeogenesis, glucose metabolism, etc, in TBU as compared to non-TBU (although statistically non-significant) generates interesting hypotheses and drives further research.<br>**Apoptosis pathway:** eliminate mycobacteria in macrophages as a defence against intracellular infection.<br>**KRAS signaling:** EGFR mutations, well-established in cancers, are influenced by the presence of pre-existing TB.<br>**Diabetes pathway:** direct and indirect impact of diabetes on development of TB.<br>**MTORC1 signaling:** inhibits MTB survival in the macrophages by inducing autophagy.<br><br>This study is the first both to report the proteomic profile of vitreous in TBU and to use shotgun proteomics in the human vitreous samples.<br><br>This study is also among the very few initial proteomic studies to be done on human vitreous sample in diseases of intraocular inflammation. Most of the studies on vitreous proteome are from animal models. The human vitreous proteomic studies, so far, have been limited to healthy eyes, glaucoma, retinoblastoma, and retinal vascular disorders (diabetic retinopathy, age related macular degeneration, retinal vein occlusion, retinopathy of prematurity, etc.).	contaminants in laboratories, which raises the origin of Keratin, type I cytoskeletal 17 in our study, whether it is a true endogenous protein (from the investigated sample) or a contaminant (from laboratory environment).<br><br>A prospective validation in future studies would add more information on the significance of this protein in ocular proteomics.<br><br>The mean age of patients in group A differed significantly from that of group B patients (group A higher than group B). But the total number of patients in each group is limited, and the age range is much wider in group B (13–65 years) than group A (25–60 years). As a result, the bias of the age influencing the proteomic profile in analysis II (groups A versus B) cannot be ruled out. Thus the comparisons made are tentative at best, and our results may be considered as a preliminary hypothesis generating data.
	Van der Colff et al. (2023)	29 biomarkers were tested on both the urine and serum samples: MMP-9, sIL-6Ra, sIL-2Ra, sIL-4R, sIL-6R, sVEGFR3, sCD30, sEGFR, sgp130, sIL-1RI, sIL-1RII, sRAGE, sTNFRI, sTNFRII, sVEGFR1, sVEGFR2, IFNγ,	Assays were run on the Bio Plex 200 platform.	The concentrations of most biomarkers studied differed markedly in urine and in serum with the majority being much lower in the urine. The findings from this study support those of a previous TB study at the same facility, also reporting significant differences in biomarker levels of serum and saliva.<br><br>Results show that it is not possible to extrapolate urine	Small sample size which may report false findings.<br><br>No concrete conclusions presented by this study, which serves as a small pilot study that will hopefully provide direction for future research.

		IL-1RA, Il-2, IP-10, MIP-1B, VEGF-A, MDC, Ferritin, A2M, CRP, Fibrinogen, SAP and Haptoglobin.		concentrations from serum values and vice versa and different biofluids therefore are not interchangeable when developing OTB biosignatures.	
Stimulation Assay	Makhoba et al. (2021)	Four-marker biosignature comprising of CD40 ligand, IL-33, IFN-γ, and SAP, which showed potential in diagnosing OTB.	Luminex Assay for 47 biomarkers in QFT supernatants.	First attempt at screening for potential biomarkers in QFT supernatants to diagnose OTB<br><br>The most clinically relevant finding of our study was a four-marker biosignature comprising of CD40 ligand, IL-33, IFN-γ, and SAP, which showed potential in diagnosing OTB as determined by an AUC of 0.80. The present study show that antigen-specific levels of the host markers investigated (with the exception of GM-CSF) were not significantly different between patients with probable or possible OTB, regardless of their HIV status.<br><br>Study participants were stratified according to their HIV status, which is a strength of this study. In HIV-negative participants, a 2-marker antigen-specific biosignature showed encouraging results while the same could not be said for the 3-marker biosignature identified in unstimulated supernatants. Conversely, in HIV-positive participants, a 5-marker antigen-specific biosignature accurately predicted 80% of HIV positive OTB and non-OTB patients while a 3-marker biosignature in unstimulated supernatants could predict 88% of HIV positive non-OTB participants.	Small sample size and further research has to be done on larger sample sizes to validate and potentially adjust for variables that may influence the results such as HIV status (alluded to above), age and other factors relevant to the recruited participants.<br><br>33.3% of the OD patients evaluated in the current study had TST positive results. This is not surprising given that our study was conducted in a high TB burden setting. Such individuals if truly latently infected with MTB, will habour T cells which will recognize the antigens used in QFT tubes, and also secrete host markers into QFT supernatants irrespective of the primary ocular diagnosis. TB host biomarker-based tests should be able to discriminate between such latently infected individuals who present with other ocular diseases and those whose cause of the ocular disease is TB if they are to be useful in high TB burden settings. This, therefore, raises the potential value of the OTB diagnostic candidate biosignatures identified in the current study.
	Alam et al. (2022)	TST-positive undifferentiated uveitis (UNK) generates a stronger monofunctional and polyfunctional (dual-cytokine) intraocular cytokine response than active OTB.	Flow cytometry	TST-positive undifferentiated uveitis (UNK) generates a stronger monofunctional and polyfunctional (dual-cytokine) intraocular cytokine response than active OTB, suggesting that the anti-TB immune response in TST-positive undifferentiated uveitis is more effective in protecting from pathogen-based tissue damage. Notably, all patients in the OTB group were treated with ATT in our study. Conversely, the UNK group, only 4 (16.7%) of whom were considered for ATT, could be representative of latent infection.<br><br>Retinal autoantigen IRBP-specific intraocular cytokine responses occurred in all cases of posterior segment uveitis, regardless of their clinical phenotype, chronicity, or TB immunoreactive status. This additional autoreactive anti-IRBP response was characteristic of both TB- and non-TB–associated	The use of single peptides for eliciting both the antimycobacterial and retinal antigen–specific responses. Thus, T cell responses to other immunodominant peptides (mycobacterial and retinal) are not covered in our data.

				intraocular inflammation.	
Serum and intraocular protein analysis	Singh et al. (2021)	Raised VEGF and decreased FGF levels in RPE cells and vitreous humour, but not in tears.	Bead-based multiplex assay, using fluorescence–encoded beads using flow cytometry (BD LSR Fortessa).	The present study showed that RPE cells even infected with a few MTB bacilli leads to switching of FGF to VEGF growth factor cytokine profile, 57 times up-regulation of VEGF and simultaneously 164 times down regulation of FGF. There was a few fold up-regulation of Ang-2 but not as drastic as VEGF.<br><br>Similar growth factor pattern changes occur in the vitreous samples of confirmed intraocular tuberculosis (IOTB) patients. FGF levels were down regulated ($p < 0.05$) in confirmed IOTB MTB PCR +ve patients compared to non-IOTB patients with MTB PCR -ve etiology. VEGF and Ang-2 levels were also elevated in IOTB patients but were not statistically significant compared to non-IOTB.<br><br>However, similar FGF and VEGF patterns were not reflected in the tear samples of clinically IOTB patients compared to non-TB uveitis patients as well as in diseased eye compared to non diseased eye of same IOTB patients. Although all the growth factors were detectable in tear samples but we did not find any statistical difference in the tear samples and no correlation was seen between vitreous and tear levels indicating the FGF and VEGF growth factor changes in the vitreous are not reflected in tear samples.	Small sample size, dilution of vitreous samples.
T-lymphocyte profiling	Hutchinson et al. (2021)	Only increased CD38 and HLA-DR expression on Mtb-specific CD4 T cells were significant to discriminate different OTB phenotypes and predict treatment response.	Poly-chromatic flow cytometry	In this study, except for CD38 and HLA-DR expression, none of the previously reported biomarkers were found to be significant in discriminating different phenotypes of ocular TB or predicting treatment response.<br><br>There is also a significant higher proportion of PPD-stimulated CD4+ IFNg+(CD27+ CD38), CD4+IFNg+(CD27-CD38) and CD4+ IFNg+(CD27+ GM-CSF IL2+ TNFa) T cells in those with bilateral uveitis compared to those with unilateral uveitis, and that the level of CD27 expression on the PPD specific CD4 T cells was higher. There was a higher proportion of these markers on the IFNg+ PPD -specific CD4 T cells in the patients who responded to ATT compared to those that did not. Taken together, this may indicate that the level of PPD-specific CD4 T cells may correlate with the activity of ocular inflammation and	There was a small sample size with only a minority of patients having undergone ATT. There was, also a negative correlation found between the degree of vitreous inflammation and the activation markers. Most likely this could be explained due to lower sample size of the study and also the fact that the study was not designed to look primarily into the activation markers and the level of intraocular inflammation. It is also possible to postulate that these activation markers are down regulated in cases with more severe intraocular inflammation.<br><br>Patients who were recruited were mainly those who were presumed (not confirmed) to have ocular TB, given the constellation of clinical findings and positivity of investigations, in which these investigations are also unable to differentiate active versus latent TB. This is an inherent challenge faced

				higher initial level of these cells may predict better treatment efficacy.<br><br>Previous studies have found reduced CD27+ expression on antigen-specific CD4 + T cells in patients with persistent active pulmonary TB, or a higher proportion of IFNg-producing MTB specific CD4 + T cells negative for/weakly-expressing CD27 in active compared to latent TB. CD27 is a CD4 + T cell memory marker, and this receptor is found to be downregulated when T cells progress from a naïve to a terminal memory stage. However, this study found no significant difference in CD27 expression on PPD specific CD4 T cells between treatment responders and non-responders, nor did it change following treatment. The findings that the MTB antigen specific cells still make IFNg after treatment is not unusual. What does seem to change is the expression of markers such as CD38, HLA-DR, and CD27, which most likely reflects some modification in the effector function of these cells. As the treatment is aimed at reducing the bacterial load rather than directly effecting immune functions this could be expected.	when performing studies on patients with ocular TB, where most are presumed cases of ocular TB since microbiological diagnostic confirmation via ocular fluids are seldom performed due to their low yield and invasiveness of test. Therefore, it is difficult to rule out other masquerades of ocular TB, such as ocular sarcoidosis and ocular syphilis, which may have different types of T cell response.
